# Comparing Ammonium and Tetraaminophosphonium Anion-Exchange Membranes Derived from Vinyl-Addition Polynorbornene Copolymers

**DOI:** 10.1021/acsaem.3c02822

**Published:** 2024-02-13

**Authors:** Jamie C. Gaitor, Ami C. Yang-Neyerlin, Danielle Markovich, Brett P. Fors, Geoffrey W. Coates, Lena F. Kourkoutis, Bryan S. Pivovar, Tomasz Kowalewski, Kevin J. T. Noonan

**Affiliations:** † Department of Chemistry, Carnegie Mellon University, 4400 Fifth Ave, Pittsburgh, Pennsylvania 15213, United States; ‡ Chemistry and Nanoscience Center, National Renewable Energy Laboratory, 15013 Denver West Parkway, Golden, Colorado 80401, United States; § School of Applied and Engineering Physics, Cornell University, Ithaca, New York 14853, United States; ∥ Department of Chemistry and Chemical Biology, Baker Laboratory, Cornell University, Ithaca, New York 14853, United States

**Keywords:** vinyl addition polynorbornenes, anion exchange membranes, fuel cell, ammonium polymers, tetraaminophosphonium polymers

## Abstract

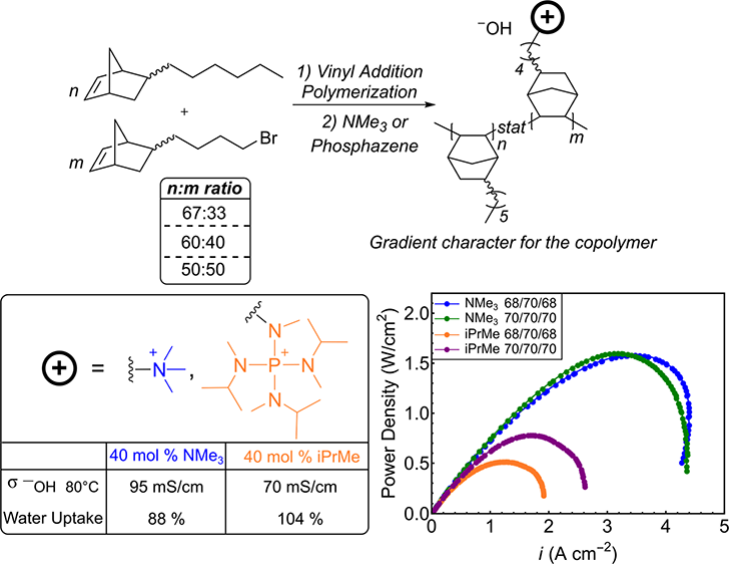

Herein, we systematically examined how composition influenced the properties of vinyl addition polynorbornene anion exchange membranes (AEMs) prepared from 5*-n*-hexyl-2-norbornene and 5-(4-bromobutyl)-2-norbornene. Copolymerization kinetics revealed that 5*-n*-hexyl-2-norbornene is consumed faster than 5-(4-bromobutyl)-2-norbornene, leading to a portion of the chain being richer in bromoalkyl groups. The alkyl halide pendants can then be converted to either trimethylammonium or tetrakis(dialkylamino)phosphonium cations through straightforward substitution with trimethylamine or a tris(dialkylamino)phosphazene. A series of cationic ammonium polymers were synthesized first, where conductivity and water uptake increased as a function of increasing ionic content in the polymer. The optimized copolymer had a hydroxide conductivity of 95 ± 6 mS/cm at 80 °C. The living polymerization of the two monomers catalyzed by a cationic *tert*-butylphosphine palladium catalyst also enabled precise changes in the molecular weight while keeping the functional group concentration constant. Molecular weight did not have a significant impact on hydroxide conductivity over the range of ∼60–190 kg/mol (*M*_n_). The optimized tetraaminophosphonium AEM had the highest conductivity for any tetraaminophosphonium polymer to date (70 ± 3 mS/cm at 80 °C). Clear phase separation and larger domains were observed for the phosphonium-based AEM compared to the ammonium at an identical composition, which is attributed to the larger occupied volume of the phosphorus cation. Fuel cell studies with the two membranes resulted in peak power densities of 1.59 and 0.79 W/cm^2^ for the ammonium and tetraaminophosphonium membrane electrode assemblies, respectively. The ammonium-based membrane was more water permeable as evidenced by water limiting current studies, which likely contributed to the improved performance.

## Introduction

**Figure 1:**
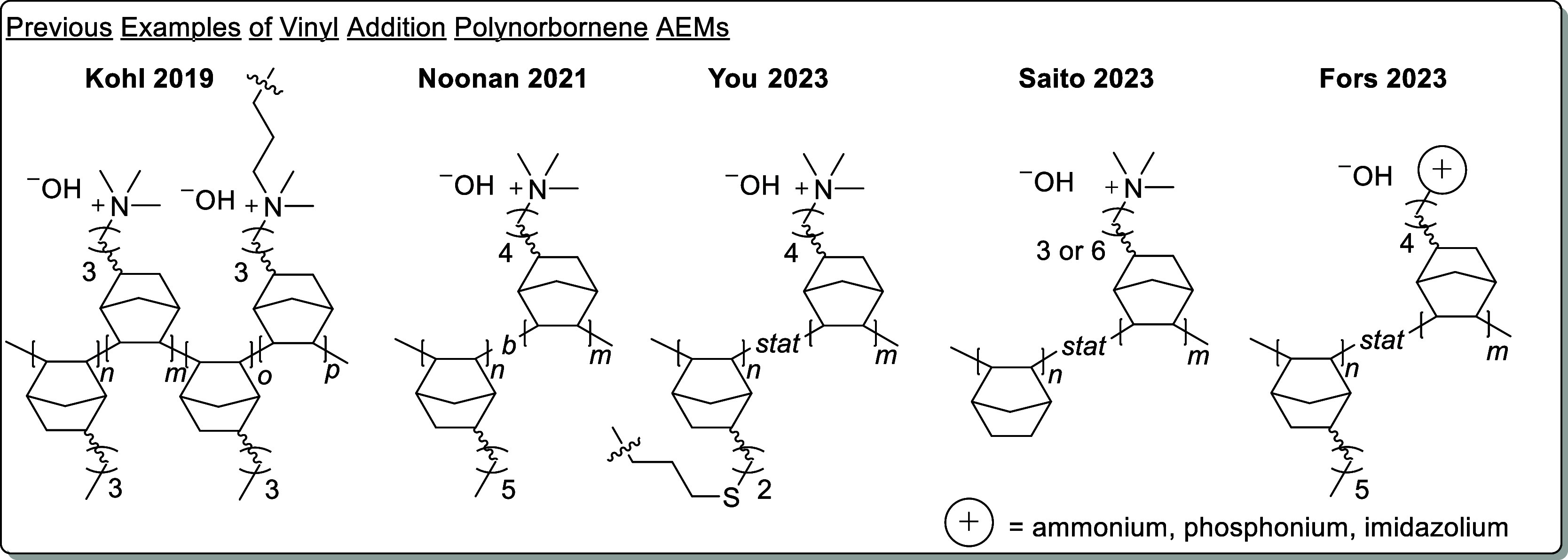
Some previously reported hydroxide-conducting polymers derived from vinyl addition polynorbornenes.^10,15−21^

Ion-exchange membranes are critical components in electrochemical devices, and over the past several years, there has been increased interest in developing hydroxide conducting anion exchange membranes (AEMs) for use in fuel cells (AEMFCs) and water electrolyzers (AEMWEs).^1−4^ Vinyl addition polynorbornenes (PNBs) have been considered for this purpose, due to the alkaline stable hydrocarbon backbone and tunable properties that can be achieved through polymerization of functional norbornene monomers.^5−8^ Trimethylammonium-based PNB copolymers have already been explored as membranes in AEMFCs and good performance has been demonstrated, with peak power densities >1 W/cm^2^.^9,10^ In addition, PNB copolymers have been explored as ionomers in both AEMFCs and AEMWEs.^11−14^ Given the promise of these materials, furthering our understanding of cationic PNB copolymers will be key to expanding on their use as AEMs (Figure 1).^10,15−21^

Herein, building from our prior work,^19^ we report on a detailed examination of statistical PNB copolymers as AEMs, gaining a better understanding of how polymer microstructure influences properties and performance. The living copolymerization of 5-*n*-hexyl-2-norbornene (NB-5-Hex) and 5-(4-bromobutyl)-2-norbornene (NB-5-BuBr) was accomplished using *t*-Bu_3_PPd(Me)Cl with lithium tetrakis(pentafluorophenyl)borate ethyl etherate as an activator. This catalyst system and several related derivatives have been demonstrated to be active for polymerization of norbornene monomers.^22−26^ Kinetic analysis revealed that the hexyl monomer is consumed faster in copolymerization, suggesting some gradient character for the copolymer, with segments of the chain being richer in bromoalkyl groups. The pendant halide groups were then converted into trimethylammonium cations, and a clear relationship between ion concentration, phase separation, and conductivity was established for the copolymers using small-angle X-ray scattering (SAXS), transmission electron microscopy (TEM), and impedance spectroscopy.

In addition, we carried out a more detailed comparison of the well-known trimethylammonium cation with a tetraaminophosphonium cation. Direct comparisons are straightforward because both cations can be attached after copolymerization of NB-5-Hex and NB-5-BuBr,^19^ ensuring the cation concentration and relative location along the chain is the same. Interestingly, we observe clear phase separation for the phosphonium copolymer at lower molar concentrations when compared to the ammonium analogue. The phase separation and tapered character of the tetraaminophosphonium copolymer is beneficial for hydroxide conductivity when compared to other related materials,^19,27−29^ as this copolymer has the highest conductivity of any tetraaminophosphonium AEM reported to date. Since the trimethylammonium and tetraaminophosphonium PNB copolymers both have excellent stability in 1 M KOH at 80 °C,^18,20^ fuel cell studies were carried out using both copolymers as membranes. Higher power density was noted for the ammonium copolymer compared to the phosphonium copolymer (1.59 W/cm^2^ vs 0.79 W/cm^2^) as well as enhanced water permeability.

## Results and Discussion

### Copolymer Synthesis and Kinetics

**Figure 2:**
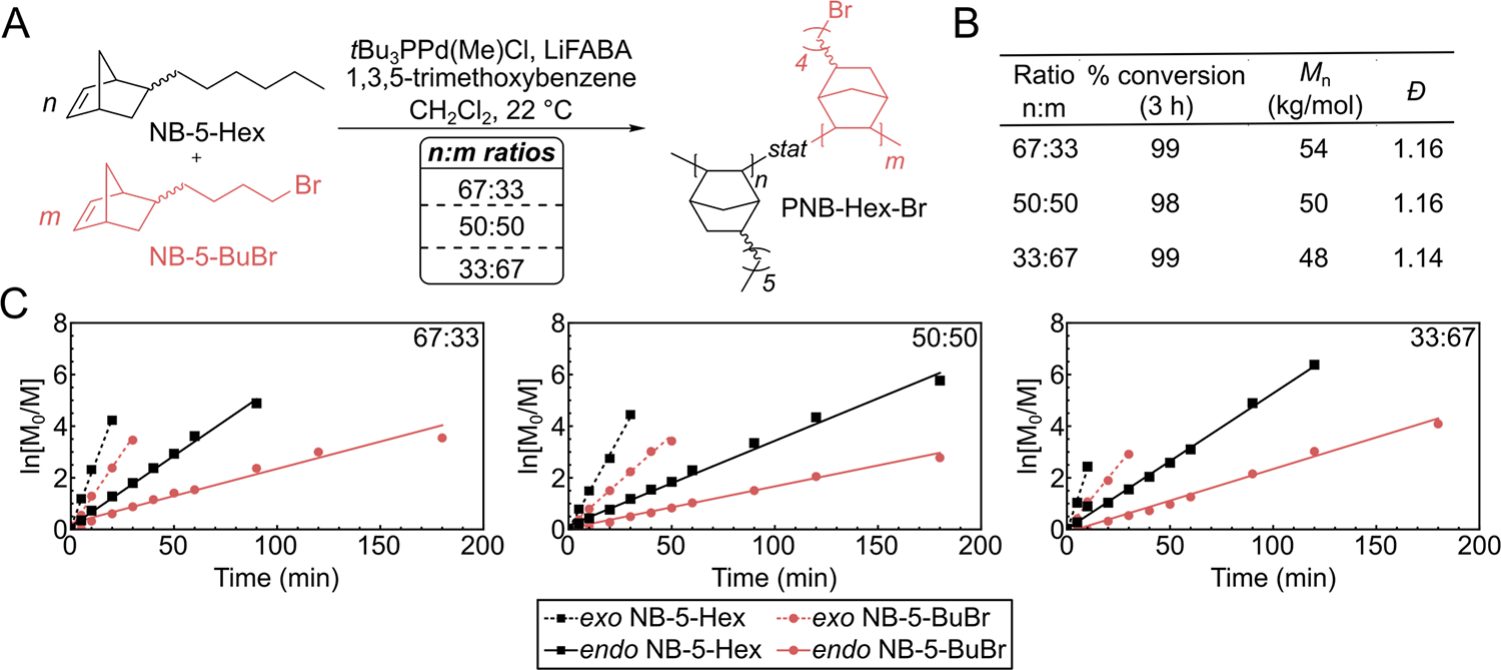
(A) General synthetic scheme for copolymerization. LiFABA stands for lithium tetrakis(pentafluorophenyl)borate ethyl etherate. The 1,3,5-trimethoxybenzene was added as an internal standard for kinetic analysis. (B) Percent conversion and molecular weight data after 3 h. (C) Semilogarithmic plots of concentration versus time for the copolymer series.

NB-5-Hex and NB-5-BuBr were synthesized similarly to prior reports and each monomer was isolated as an ∼80:20 mixture of *endo*:*exo* stereoisomers.^30,31^ When polymerizing stereoisomer mixtures in vinyl addition polymerization, *exo* isomers are typically enchained faster than the *endo* isomers, leading to a down-chain *exo*-gradient.^22,25,32,33^ Cationic palladium^18,34^ and neutral nickel catalysts^31^ have been used for polymerization of bromoalkylnorbornenes, and here, *t-*Bu_3_PPd(Me)Cl activated with lithium tetrakis(pentafluorophenyl)borate was used as the catalyst for copolymerization (Figure 2A). Monomer reactivity was examined by combining NB-5-Hex and NB-5-BuBr in molar ratios of 67:33, 50:50, and 33:67 (NB-5-Hex:NB-5-BuBr) with 0.2 mol % cationic Pd catalyst and 1,3,5-trimethoxybenzene as an internal standard in CH_2_Cl_2_ (Figure 2B,C). Implementation of the terminal model of copolymerization was considered to calculate reactivity ratios, but the four kinetically distinct monomers present in the reaction mixture make this type of analysis difficult.^32^ Aliquots were periodically removed from the reaction mixture and analyzed using gas chromatography–mass spectrometry (GC-MS) to determine relative rates of monomer consumption (Figure 2C).

All copolymerizations were near complete (>98%) after 3 h (Figure 2B). Monomer stereochemistry had the anticipated effect on initial composition, where copolymers were comprised of >75% *exo* repeat units after 5 min despite the lower concentration of the *exo* isomer relative to *endo* in the mixture (Table S2). Semilogarithmic plots of concentration over time show that, independent of the ratio of each monomer in the initial feed, the *exo* NB-5-Hex monomer was enchained the fastest, followed by *exo* NB-5-BuBr, then *endo* NB-5-Hex, and finally *endo* NB-5-BuBr (Figure 2C). These observations qualitatively suggest that the copolymer composition is richer in the insulating monomer at the outset, with gradual enrichment of the functional monomer over the course of the polymerization. Altogether, the results suggest some gradient character of the hexyl and butyl bromide groups along the polynorbornene chain. Throughout the text, the synthesized copolymers are described in terms of the polymer mainchain (PNB), the insulating hexyl chain (Hex), the pendant functional group, and the counterion when applicable (neutral copolymers = PNB-Hex-Br; ionic copolymers = PNB-Hex-NMe_3_[X] and PNB-Hex-iPrMe[X], where NMe_3_ refers to the trimethylammonium cation, iPrMe refers to the tetraaminophosphonium cation, and X = Cl^–^, Br^–^, or ^–^OH).

Two other reports^10,16^ have appeared on copolymerization of alkylnorbornenes and bromoalkylnorbornenes for postfunctionalization to obtain AEMs. Kohl and co-workers carried out a copolymerization of 5-butylnorbornene and NB-5-BuBr with (^*i*^Pr_3_P)Pd(η^3^-allyl)Cl,^16^ while Saito and co-workers copolymerized NB-5-Hex, norbornene, and bromoalkylnorbornenes with (SbPh_3_)_2_Ni(C_6_F_5_)_2_.^10,35^ In both reports, the copolymers were denoted as random, but an in-depth kinetic analysis of the copolymerization was not included. It is difficult to precisely compare those examples with the data obtained here since different catalysts and monomers were used in all instances.

### Synthesis and Thermal Characterization of PNB-Hex-NMe_3_ Copolymers

Following kinetic analysis, PNB-Hex-Br copolymers were synthesized for conversion to AEMs. The targeted molar ratios of NB-5-Hex and NB-5-BuBr (67:33, 60:40, and 50:50) were selected to keep the ion-exchange capacity (IEC) within a reasonable range (entries 1–3 in Table 1). Attempts to incorporate more NB-5-BuBr and further increase IEC led to AEMs which swelled excessively or dissolved in water.

**Table 1:** Properties of PNB Copolymers

entry	NB-5-Hex:NB-5-BuBr	*M*_n_^a^ (*Đ*) kg/mol	cation	IEC_theo_^b^ (mmol/g)	IEC^c^ (mmol/g)	WU^d^^–^OH (%)	λ^e^	σ ^–^OH (mS/cm)^f^	
								22 °C	80 °C
1	67:33	59 (1.19)	NMe_3_	1.69	1.38	45	18	27 ± 1	57 ± 6
2	60:40	59 (1.18)	NMe_3_	1.97	1.91	88	26	45 ± 3	95 ± 6
3	50:50	79 (1.13)	NMe_3_	2.34	2.19	109	28	44 ± 1	104 ± 5
4	60:40	105 (1.20)	NMe_3_	2.00	2.03	93	25	46 ± 3	98 ± 6
5	60:40	192 (1.33)	NMe_3_	2.01	1.99	103	29	47 ± 2	100 ± 5
6	67:33^g^	80 (1.13)	iPrMe	1.25	1.12	59	29	26 ± 2	52 ± 3
7	60:40	59 (1.18)	iPrMe	1.38	1.32	103	43	32 ± 2	70 ± 3

a Molecular weights of PNB-Hex-Br copolymers were determined versus polystyrene standards at 40 °C with tetrahydrofuran doped with 10 mM lithium bis(trifluoromethanesulfonyl)imide as the eluent.

b Theoretical IEC values were calculated based on the concentration of alkyl halide groups estimated from ^1^H NMR spectroscopy (Figures S6 and S7).

c Experimental IEC values were measured using standard back-titration methods.^28^

d Water uptake (WU) was determined gravimetrically for each film in the ^–^OH form.

e Hydration values (λ) were determined according to the equation λ = [1000 × WU]/[IEC × 18].

f Conductivity values were determined using electrochemical impedance spectroscopy with the reported values as an average of three or more trials (error is standard deviation).

g Data obtained from ref (19).

**Figure 3:**
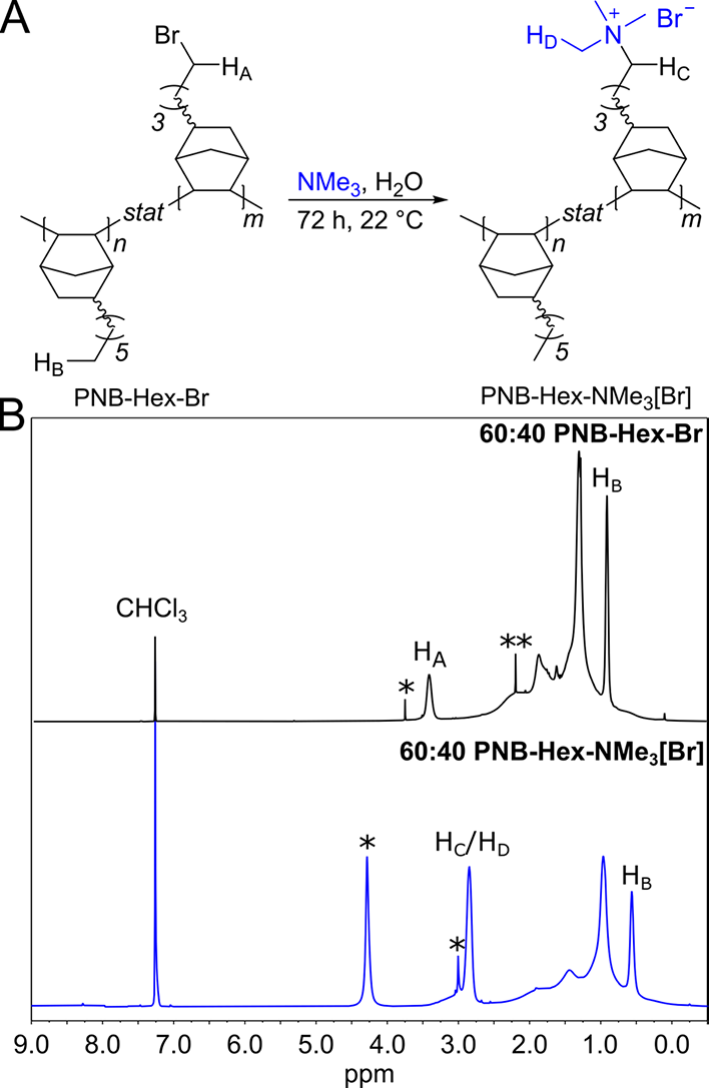
(A) General synthetic scheme for PNB-Hex-NMe_3_[Br] synthesis. (B) Top: ^1^H NMR spectrum of 60:40 PNB-Hex-Br collected in CDCl_3_ (500 MHz, 22 °C). The signal with a * corresponds to residual C_2_H_4_Cl_2_. The signal with a ** corresponds to residual acetone. Bottom: ^1^H NMR spectrum of 60:40 PNB-Hex-NMe_3_[Br] collected in CDCl_3_/CD_3_OD (50% v/v). The signals with an asterisk correspond to residual protio CD_3_OD (∼3 ppm) and H_2_O (∼4.27 ppm).

Polymerizations were initiated using 0.2 mol % of the cationic Pd catalyst to obtain copolymers with a degree of polymerization (DP) of ∼500, and the polymers were isolated in >90% yield in all instances. The relative ratios of the two enchained norbornenes in the copolymers can be estimated using ^1^H NMR spectroscopy by integration of the methylene bromide signal (H_A_) from NB-5-BuBr and the methyl signal (H_B_) from NB-5-Hex (representative spectrum shown in Figure 3). For all targeted compositions, the ratios of the comonomers were within 3% of the target value (Figure S6). The *M*_n_ values ranged from 59–79 kg/mol for the series (entries 1–3, Table 1), which is above the reported chain entanglement molecular weight for poly(5-*n*-hexylnorbornene).^36^ Free-standing thin films of the PNB-Hex-Br copolymers were then obtained by drop-casting CHCl_3_ solutions in a stainless-steel Petri dish.

The neutral PNB-Hex-Br films were immersed in 25 wt % NMe_3(aq)_ for 72 h to convert the pendant bromide groups into trimethylammonium groups (Figure 3A).^19^ The resultant PNB-Hex-NMe_3_[Br] copolymers were soluble in *n*-propanol, and could be dissolved in a 1:1 mixture of CDCl_3_ and CD_3_OD for ^1^H NMR analysis (Figure 3B). It should be noted that when the ^1^H NMR spectrum was referenced to CHCl_3_ (7.26 ppm), the shift for the protio CD_3_OD signal was upfield from the expected value (noted with an asterisk in bottom of Figure 3B). The methyl and methylene signals for the ammonium group (H_C_ and H_D_) are not well resolved and overlap with those of the protio CD_3_OD solvent. The relative integration of H_D_:H_B_ is a little lower than expected (∼75%), which we suspect is partially a consequence of the overlap with the solvent signal and poor solvation of the polymer in the CDCl_3_:CD_3_OD mixture. The diagnostic methylene signal for PNB-Hex-Br was absent in the PNB-Hex-NMe_3_[Br] sample, suggesting that quaternization was effective. To provide further confirmation of the substitution, titration experiments to determine the IEC were carried out. Experimental IEC values were within 20% of the theoretical value in all instances (entries 1–5 in Table 1), suggesting that the functionalization with NMe_3_ was near the targeted percentage for all synthesized copolymers using this approach.

Thermogravimetric analysis (TGA) was performed on ∼5 mg of PNB-Hex-NMe_3_[Cl] samples after IEC measurements. The onset of decomposition (*T*_d_ 5%) for the series was from 200–205 °C (Figure S13), close to the *T*_d_ 5% of an ammonium-functionalized statistical copolymer from our prior report (210 °C).^19^ A two-step decomposition is observed, where the first step is a 15% mass loss likely due to cation degradation, while the remainder of the mass loss occurs in the second step and is attributed to polymer backbone degradation (Figure S13).

### SAXS and TEM

**Figure 4:**
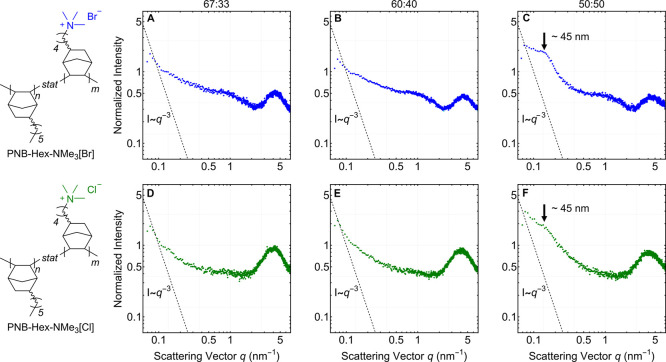
Invariant normalized SAXS scattering patterns for the PNB-Hex-NMe_3_[X] copolymers acquired in tall, narrow-slit collimation at 22 °C. Panels A–C correspond to X = ^–^Br and panels D–F correspond to X = ^–^Cl. The dashed diagonal lines were included as reference to ideal Porod scaling which, for the narrow-slit collimator, can be approximated as *I* ∼ *q*^–3^.^38^ The molar concentrations of NB-5-Hex and the ammonium-functionalized norbornene in the copolymer (ratio of *n*:*m*) are noted at the top of the plots.

Small-angle X-ray scattering (SAXS) was used to probe the nanoscale organization of the dry ammonium copolymers with both Br^–^ and Cl^–^ counterions (Figure 4A–F). Low *q* peaks are absent in the scattering patterns for the 67:33 and 60:40 copolymers but, a slope change in the low *q* range (<0.5 nm^–1^) was interpreted as weak separation between insulating and ionic domains (Figure 4A,B,D,E). A slightly larger deviation from Porod scaling (*I* = *q*^–3^) was noted for the bromide membranes, which might suggest more distinct boundaries between insulating and ionic segments with the heavier counterion (Figure 4A,B).^37^ A broad peak between *q* = 0.1–0.2 nm^–1^ was observed for the 50:50 copolymer corresponding to phase separated ∼45–50 nm domains, independent of the counterion (Figure 4C,F). This feature appears between the length scales observed for our previously synthesized diblock and triblock copolymer (65 and 36 nm, respectively)^19^ and indicates clear phase separation. Since consumption of NB-5-BuBr is slower in these copolymerizations leading to some gradient character for the polymer microstructure, it is not surprising that nanophase separation is observed with a higher incorporation of the functional monomer. In the mid-*q* range (∼1 nm^–1^), scattering intensity increases for all PNB-Hex-NMe_3_[Br] membranes which is attributed to short-range aperiodic clustering of the ionic groups (Figure 4A–C).^19^ There are no discernible increases in scattering intensity for the analogous chloride membranes in this range. A broad peak corresponding to the wide angle scattering amorphous halos of all copolymers was observed near ∼5 nm^–1^, which is more pronounced with the ^–^Cl counterion (Figure 4D–F).

**Figure 5:**
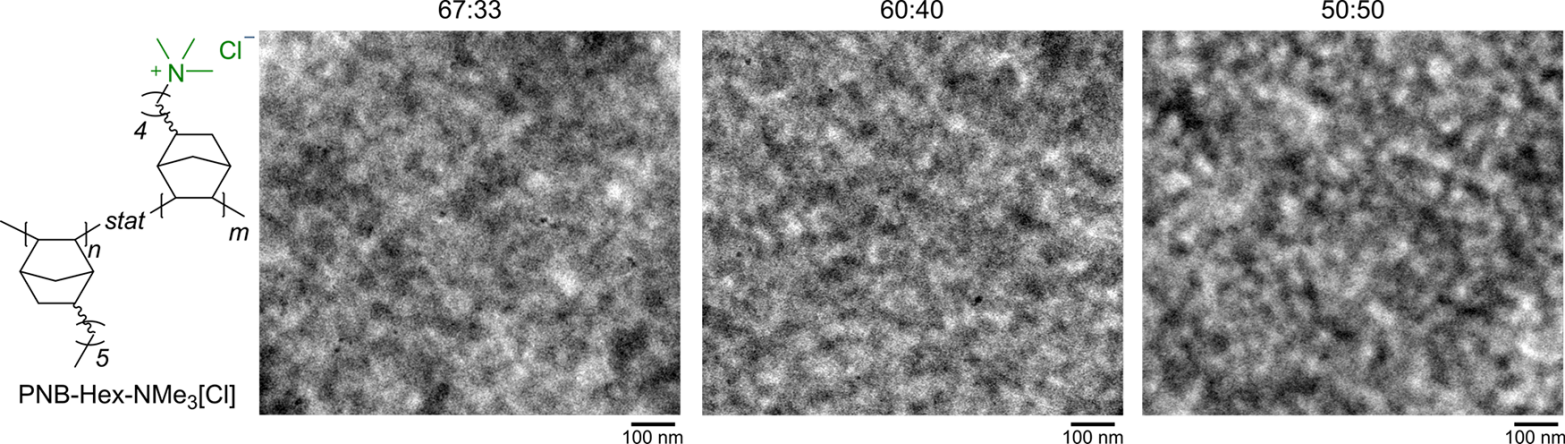
Cryo-TEM images of the 67:33, 60:40, and 50:50 PNB-Hex-NMe_3_[Cl] copolymers (left to right). Microphase separation is observed on the 40–70 nm length scale, as determined from fast Fourier transforms of the images.

Cryogenic transmission electron microscopy (cryo-TEM) was carried out to provide additional information about the morphologies of the PNB-Hex-NMe_3_[Cl] copolymers. Roughly 30 nm thick cross sections were prepared using cryo-ultramicrotomy. All membranes were free-standing films with good mechanical integrity, enabling straightforward sample preparation and analysis. We attribute the light sections of the images to the ionic domains and the dark regions to the dense aliphatic content related to the polymer main chain (Figure 5).^39^ The contrast between these regions indicates that there is clear phase separation between insulating and ionic domains with moderate continuity. Ionic domains on the order of ∼40–70 nm are visible in cryo-TEM images regardless of the cation concentration (Figure 4). Stronger contrast was noted for the 50:50 copolymer, suggesting stronger microphase separation, which is in agreement with the SAXS profile.

### Water Uptake (WU) and Hydroxide Conductivity

The PNB-Hex-NMe_3_[OH] membranes were obtained by immersion of the halide form copolymers in 1 M KOH at 80 °C for 48 h. Gravimetric analysis was used to determine the WU at room temperature by comparing the mass of hydrated ^–^OH films to dry Cl^–^ films. WU increased as a function of increased cation incorporation (45–109%, entries 1–3 in Table 1). The 67:33 PNB-Hex-NMe_3_[OH] membrane had the lowest WU (45%) although this was 15% higher than our previous report which may be due to slight differences in cation incorporation.^19^ The 60:40 and 50:50 PNB-Hex-NMe_3_[OH] membranes had WUs of 88 and 109%, respectively.

Electrochemical impedance spectroscopy was performed to determine the hydroxide conductivity of each AEM. Conductivity (σ) at 80 °C for the 67:33 PNB-Hex-NMe_3_[OH] AEM was 57 mS/cm (entry 1 in Table 1), which is within error of the analogous copolymer from our prior work.^19^ The σ_80°C_ for 60:40 PNB-Hex-NMe_3_[OH] (95 ± 6 mS/cm) was markedly higher than that for the 67:33 sample (57 ± 6 mS/cm). Increasing the ionic content further to 50 mol % led to a minor increase in σ_80°C_ (104 ± 5 mS/cm) but a 20% increase in WU. The conductivity values for the 50:50 PNB-Hex-NMe_3_[OH] were similar to our 67:33 tetrablock PNB-Hex-NMe_3_[OH] AEM copolymer synthesized previously.^19^ The WU for the 50:50 statistical copolymer was 24% higher than that of the previous tetrablock copolymer (85%), which is expected considering the higher ion exchange capacity (2.19 mmol/g vs 1.67 mmol/g). Comparing all three statistical copolymer compositions, the 60:40 PNB-Hex-NMe_3_[OH] copolymer had the best balance of WU (<100%) and conductivity (σ_80°C_ = 95 ± 6 mS/cm).

Conductivities seem to be within expectation when comparing to the prior reports on random PNB-based copolymer AEMs.^10,16^ The conductivity of the 67:33 PNB-Hex-NMe_3_[OH] AEM reported here is reasonably close to the conductivity of a similar polymer reported by Saito, Kim, and co-workers.^10^ In that work, they synthesized a NB-5-Hex, norbornene, and 5-bromopropylnorbornene terpolymer, which was converted into a trimethylammonium-based AEM (σ_80°C_ = 68 mS/cm).^10^ The conductivity of the PNB-Hex-NMe_3_[OH] copolymers described here are roughly half compared to the random copolymers reported by Kohl and co-workers which is not surprising since the IEC’s of the copolymers reported here are lower (1.38−2.19 mmol/g).^16^ In Kohl’s work, very high IEC’s were obtained (3.48–3.58 mmol/g) by partial cross-linking of the material via quaternization with diamines.^16^

### Effect of Molecular Weight

Given that the polymerization process is living,^22,23^ molecular weight in these reactions can be precisely controlled. Two additional 60:40 PNB-Hex-Br copolymers were synthesized with DP targets of 1000 and 2000 to determine whether changes in molecular weight impact the WU and ^–^OH conductivity of the membranes. The *M*_n_ range for the three copolymers was ∼60–190 kg/mol (entries 2, 4, and 5 in Table 1), and they were all within 1% of the target NB-5-BuBr ratio according to ^1^H NMR spectroscopy (Figures S6 and S7). IEC values of these copolymers were very close to the theoretical values (entries 4 and 5 in Table 1). The WU for the DP 1000 NMe_3_ polymer and DP 2000 NMe_3_ polymer (93 and 103%) are slightly higher than that of the DP 500 NMe_3_ polymer (88%) which we suspect is related to the slight differences in experimental IEC. Although there are minor differences in WU and IEC, the ^–^OH conductivity for these three copolymers were within error (95–100 mS/cm at 80 °C) suggesting changes in this molecular weight range did not significantly impact transport.

### Phosphonium-Functionalized PNBs

**Figure 6:**
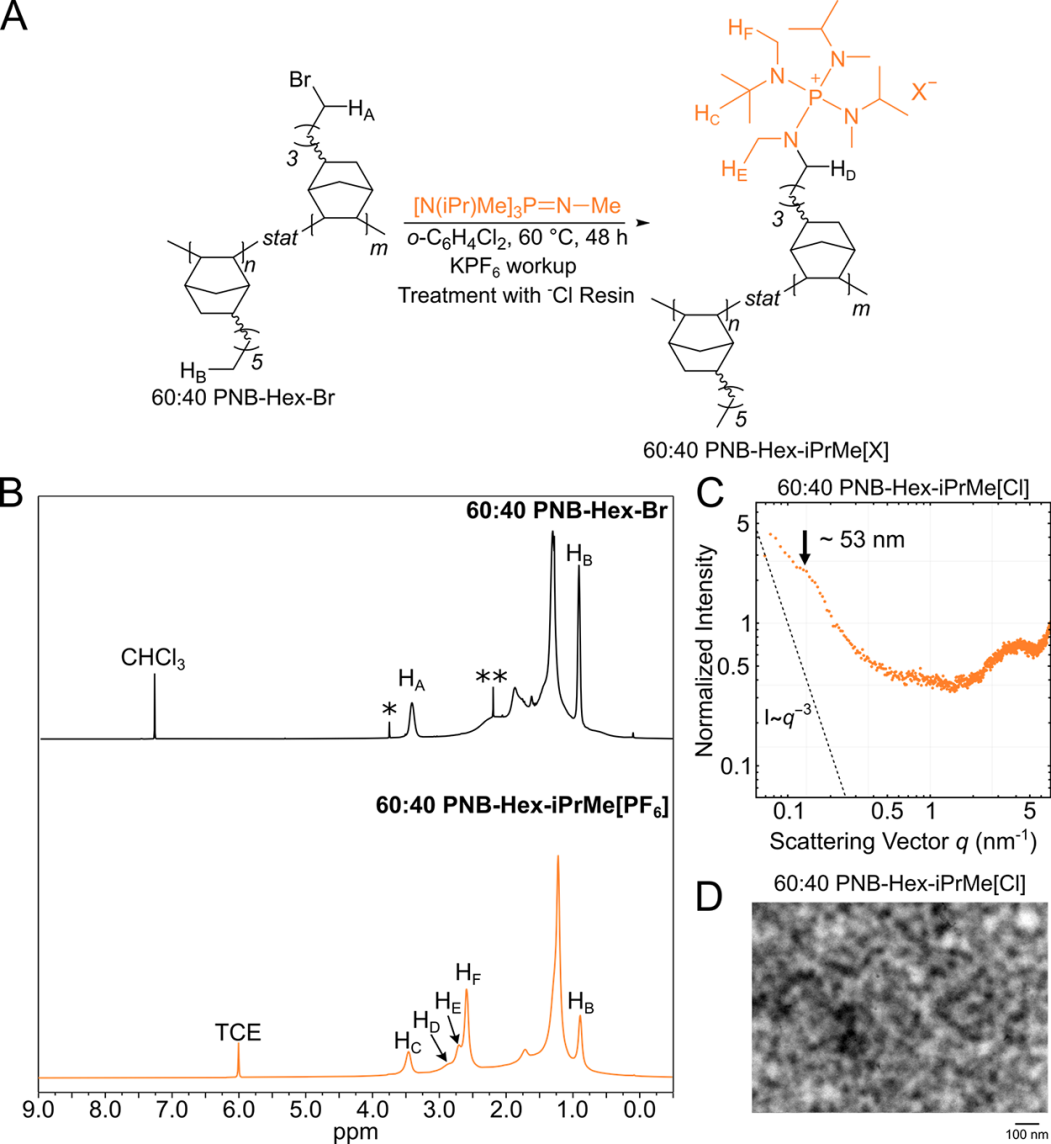
(A) General synthetic scheme for PNB-Hex-iPrMe[PF_6_] synthesis. (B) Top: ^1^H NMR spectrum of 60:40 PNB-Hex-Br collected in CDCl_3_. The signal with a * corresponds to residual C_2_H_4_Cl_2_. The signal with a ** corresponds to residual acetone. Bottom: ^1^H NMR spectrum of 60:40 PNB-Hex-iPrMe[PF_6_] collected in 1,1,2,2-tetrachloroethane-*d*_2_ (TCE-*d*_2_). (C) SAXS pattern for 60:40 PNB-Hex-iPrMe[Cl]. (D) Cryo-TEM image of 60:40 PNB-Hex-iPrMe[Cl]. Roughly 30 nm thick cross sections were prepared using cryo-ultramicrotomy and microphase separation was observed on the 60–100 nm length scale as determined from the fast Fourier transform of the image.

The PNB-Hex-Br copolymers can be converted into a tetraaminophosphonium AEM through reaction with a tris(dialkylamino)phosphazene, similar to our prior report.^19^ These resonance stabilized phosphorus cations are exceptionally alkaline stable.^27,29,40−42^ The PNB-Hex-Br copolymers can be dissolved in 1,2-dichlorobenzene and mixed with an excess of [N(iPr)Me]_3_P=N–Me at 60 °C for 48 h (Figure 6A), followed by a KPF_6_ workup to obtain the desired ionic copolymer (Supporting Information).

The conversion of the alkyl halide into the corresponding tetraaminophosphonium group can be confirmed by using NMR spectroscopy (Figure 6B). Broad signals in the ^1^H spectrum appear between 3.0–2.4 ppm, which correspond to the N–CH_3_ and N–CH_2_ groups that are directly attached to the phosphorus atom (H_D_, H_E_, and H_F_ in Figure 6B). In addition, the ^31^P{^1^H} NMR signal at 44 ppm provides additional evidence for the tetraaminophosphonium cation appended to the chain as this chemical shift is in line with expectation for these ionic compounds (Figure S10).^42^ Comparison of the integration values for H_D_, H_E_, and H_F_ to the methyl signal of the hexyl chain (H_B_) indicates that the extent of functionalization is nearly quantitative (Figure S9). Moreover, the measured IEC value for the 60:40 copolymer is within 2% of the theoretical value. The synthesized PNB-Hex-iPrMe[Cl] copolymers are soluble in 1,2-dichloroethane and can be drop-cast directly from this solvent to afford free-standing films.

Thermogravimetric analysis (TGA) was performed on ∼5 mg of 60:40 PNB-Hex-iPrMe[Cl], and the onset of decomposition (*T*_d_ 5%) for the phosphonium copolymer was 279 °C (Figure S13), which is nearly 70 °C higher than that of the corresponding ammonium copolymer. A two-step decomposition is observed just like in the ammonium copolymer case. The first step (∼10% loss) is likely due to cation degradation (Figure S13), which could be due to nucleophilic attack of the Cl^–^ anion on a methyl group. The second step is likely polymer degradation, as evidenced by the large mass loss above 400 °C (Figure S13). The TGA analysis suggests improved stability for the phosphonium cation as compared to the ammonium analogue in the presence of the nucleophilic Cl^–^.

Nanoscale features of PNB-Hex-iPrMe[Cl] were probed using SAXS. A low *q* peak was absent in the scattering pattern for the 67:33 PNB-Hex-iPrMe[Cl] copolymer (Figure S12). A change in slope was noted in this region, just like in the ammonium derivative (Figure 4), suggesting weak phase separation. For the 60:40 PNB-Hex-iPrMe[Cl], a broad peak is observed at *q* ∼ 0.12 nm^–1^, indicating phase separation (Figure 6C). This peak is absent in the 60:40 PNB-Hex-NMe_3_[Cl] analogue. Although the mole fractions of the two cations are identical in these instances, the weight fractions are markedly different, with the phosphonium cation weighing ∼3.5× more than the ammonium. Assuming similar densities, this means that the occupied volume of the phosphonium fraction is much larger, which likely contributes to the observation of phase separation at lower molar concentration. Further confirmation of the strong phase separation in 60:40 PNB-Hex-iPrMe[Cl] was evidenced by the sharp contrast between the hydrophilic and hydrophobic regions in the cryo-TEM images (Figure 6D). The TEM data also revealed a slightly larger ionic domain length scale when compared to that of the 60:40 PNB-HexNMe_3_[Cl] (∼40–70 nm vs ∼60–100 nm), suggesting an increase in ionic domain size with larger cations.

The WU and hydration values increase with higher concentration of phosphonium cation, as expected (entries 6 and 7 in Table 1).^19^ The conductivity values at 22 and 80 °C for the 60:40 PNB-Hex-iPrMe[OH] were 32 and 70 mS/cm, respectively (entry 7 in Table 1), which are the highest values for a tetra(dialkylamino)phosphonium-functionalized AEM to our knowledge.^19,27−29^ The gradient character of the copolymer is likely a direct benefit here. The increased IEC, along with the phase separation observed in SAXS and TEM are both contributors to the improved ^–^OH transport. This material also outperforms our previous phosphonium pentablock copolymer, where swelling with well-defined blocks was severe (150%). The SAXS pattern for the pentablock shows very strong phase separation (Figure S12), which likely contributes to the very high WU, highlighting the benefits of the statistical copolymer approach with bulky cations.

The WU for the 60:40 PNB-Hex-iPrMe[OH] was 15% higher than the analogous NMe_3_ copolymer, with nearly twice the hydration number (43 vs 26, Table 1). These results are consistent with our prior work,^19^ and highlight the challenge of further increasing IEC with heavier cations without swelling. The conductivity values for the 60:40 PNB-Hex-iPrMe[OH] are ∼25% lower than the comparable NMe_3_ membrane which can be partially attributed to the reduced IEC (entries 2 and 7 in Table 1).

### Device Measurements

**Figure 7:**
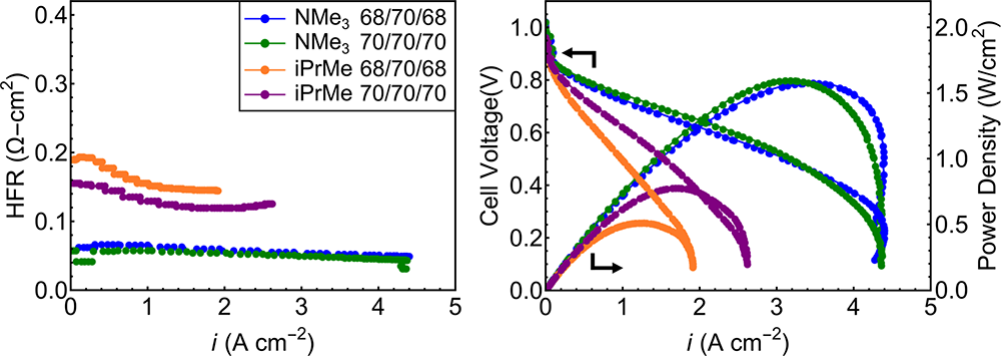
In both cases, the 60:40 ammonium and phosphonium copolymers are abbreviated as NMe_3_ and iPrMe, respectively. Left: HFR values for the membrane electrode assemblies (MEAs). Right: H_2_/O_2_ polarization curves and power density curves plotted as a function of current density. Absolute pressure for both electrodes = 131 kPa, and the inset values correspond to anode-gas-dewpoint/cell temperature/cathode-gas-dewpoint in °C.

Fuel cell device measurements were then carried out for the 60:40 ammonium and phosphonium AEMs (Figure 7). The ionomer used in each electrode was a perfluorinated anion exchange material (PFAEM) developed at the National Renewable Energy Laboratory,^43^ so water limiting current studies could be compared directly to a prior report.^44^ PtRu/C was used at the anode with a target PtRu loading of 0.8 mg/cm^2^, and Pt was used at the cathode with a target Pt loading of 0.4 mg/cm^2^. The ^–^OH form of the ammonium and phosphonium films were ∼33 and 37 μm thick, respectively, and could be compressed between the electrodes without damage during device fabrication. For both cells, anode and cathode dew points were identical and device tests were carried out at dew points of 68 and 70 °C. Cell operation temperature was 70 °C in each instance, and absolute pressure at both electrodes was set to 131 kPa. The break-in procedure was carried out by applying a constant voltage of 0.5 V until current density plateaued (∼1 h).^45^ Further procedural details are included in the Supporting Information, and experiments were carried out similarly to prior reports.^44−46^

Polarization curves were collected for both cells, and high frequency resistance (HFR) measurements were collected during cell operation (Figure 7 left). HFR for the cell containing the phosphonium AEM (∼0.14 Ω cm^2^) was nearly triple that of the ammonium-based cell (∼0.05 Ω cm^2^) at similar film thickness, in agreement with the lower ^–^OH conductivity from ex situ impedance measurements for the phosphonium membranes. Both membranes showed slightly decreasing HFR with increasing current density (Figure 7 left), suggesting that water generation at the anode played a role in improving the cell hydration level.

The peak power density for the 60:40 PNB-Hex-NMe_3_[OH] cell approached 1.59 W/cm^2^ with current density = 3.51 A/cm^2^ (Figure 7), independent of gas humidification level (between 68 and 70 °C dew points). While anode and cathode dew points had little if any impact on the ammonium-based cell, it did have a marked impact on performance of the phosphonium-based cell likely due to lower water transport, as highlighted later. For the 60:40 PNB-Hex-iPrMe[OH] MEA, 68 °C dew points at the anode and cathode produced a peak power density of 0.52 W/cm^2^ at a current density of 1.29 A/cm^2^ (Figure 7). Increasing the dew point to 70 °C proved beneficial, resulting in a peak power density of 0.79 W/cm^2^ at a current density of 1.74 A/cm^2^.

**Figure 8:**
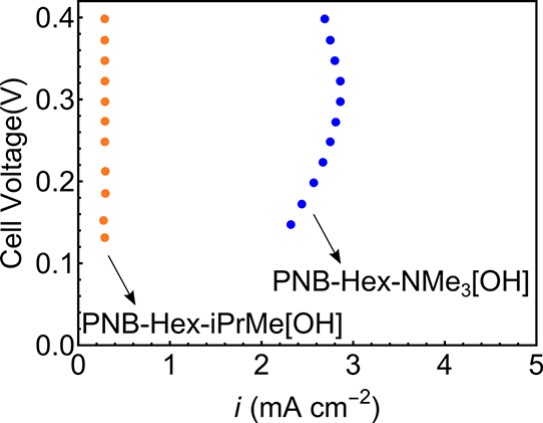
Water limiting current studies for 60:40 PNB-Hex-NMe_3_[OH] (blue) and 60:40 PNB-Hex-iPrMe[OH] (orange). 100% anode relative humidity, 0% cathode relative humidity, 70 °C cell temperature, and 131 kPa absolute pressure.

Proper balance of water between anode and cathode during fuel cell operation is critical toward achieving high performance and durability in alkaline fuel cells.^44,47−50^ Water limiting current measurements have emerged as a method to isolate the role of water flux through the membrane in the membrane-electrode assembly (MEA),^44^ and were implemented here to compare the water permeability of ammonium and phosphonium AEMs. In this experiment, the cell temperature was held at 70 °C, cathode relative humidity (RH) was 0% with a gas flow rate of 200 mL/min, anode RH was 100% with a gas flow rate of 500 mL/min, and an absolute pressure of 131 kPa was applied to both electrodes. The limiting current density for the NMe_3_ system was 2.25–2.75 A/cm^2^, while for the phosphonium cell, the limiting current was roughly 0.25 A/cm^2^ (Figure 8) suggesting major differences in water permeability. This result is interesting considering that the tetraaminophosphonium membrane has a higher gravimetric WU than the ammonium membrane, and the hydration values for the phosphonium copolymer are much larger (entries 2 and 7 in Table 1). We hypothesize that the more hydrophilic ammonium cation improves water mobility through the membrane as compared to the phosphonium analogue and suggests an opportunity to improve phosphonium cations through structural design.

H_2_ crossover (*i*_H2_, mA/cm^2^) was also measured for both materials prior to the break-in procedure and after the durability test (Figure S15). For these measurements, a pure stream of H_2_ was used at the anode while N_2_ was used at the cathode. The initial hydrogen crossover was 6 mA/cm^2^ for the NMe_3_ cell and 8 mA/cm^2^ for the iPrMe cell. After the durability study was complete, the observed H_2_ crossover value for the NMe_3_ membrane increased to 11 mA/cm^2^ while the value for the iPrMe material was unchanged. This increase may be meaningful but is not fully understood. Each of these measurements is relatively close when compared to either the observed HFR and/or the water limiting current measurements.

A short-term fuel cell durability assessment was then carried out at a constant current density of 600 mA/cm^2^ to compare the cell potential and HFR for the ammonium (114 h) and phosphonium (72 h) MEAs (Figure S15). Potential at the outset was ∼0.7 V for each cell, with relatively similar decay rates for both systems. The HFR for both cells remained relatively stable, suggesting that any loss of performance was most likely not due to membrane degradation, making it difficult to compare the stabilities of the two cations.

## Conclusions

Vinyl addition polymerization was used to prepare a series of statistical copolymers from NB-5-Hex and NB-5-BuBr that were then functionalized with trimethylammonium and tetraaminophosphonium cations. Analysis of the copolymerization kinetics revealed some gradient character for the functional units along the chain. As the concentration of ions increased in the ammonium series, a sharper phase separation between insulating and ionic regions was observed. Conductivity and WU also increased with increasing IEC, although WU became an issue with a 1:1 molar ratio of the two monomers. Molecular weight was examined as an optimization parameter over the range 60–190 kg/mol, but hydroxide conductivity of the ammonium membranes did not change significantly over this molecular weight range.

Functionalization of the polynorbornene with a tetraaminophosphonium cation led to the highest hydroxide conductivity for this type of phosphonium AEM to date. The section of the chain rich in bulky ionic groups was likely beneficial for performance as it facilitates phase separation and improved transport. Phase separation between hydrophilic and hydrophobic domains occurred at lower molar concentrations for the phosphonium AEM compared with the analogous ammonium. The ammonium and phosphonium copolymers were then compared as membranes in an alkaline fuel cell test, where higher peak power density and improved water permeability was noted for the ammonium-based cell as compared to the phosphonium. Further investigation will be aimed toward understanding in situ water transport dynamics for these materials while also trying to decrease the hydrophobicity of the phosphonium cation to improve water mobility.

## Supporting Information Available

NMR spectra, GPC traces, gas chromatograms (GC), thermal analysis (TGA), sample Nyquist plots, fuel cell durability, and H_2_ crossover (PDF)
