# Supplementary material for: Comparing Ammonium and Tetraaminophosphonium Anion-Exchange Membranes Derived from Vinyl-Addition Polynorbornene Copolymers
Source: ACS Appl Energy Mater. 2024 Feb 13;7(4):1517–26. doi: 10.1021/acsaem.3c02822 (PMC10900180; doi:10.1021/acsaem.3c02822)
Supplement: Supplementary file 1 — ae3c02822_si_001.pdf [file ae3c02822_si_001.pdf]

Supporting Information

Comparing Ammonium and Tetraaminophosphonium Anion-Exchange Membranes Derived from Vinyl-Addition Polynorbornene Copolymers

*Jamie C. Gaitor,[a] Ami C. Yang-Neyerlin,[b] Danielle Markovich,[c] Brett P. Fors,[d] Geoffrey W. Coates,[d] Lena F. Kourkoutis,[c] Bryan S. Pivovar,[b] Tomasz Kowalewski,[a] Kevin J. T. Noonan[a]**

[a]Department of Chemistry, Carnegie Mellon University, 4400 Fifth Ave, Pittsburgh, Pennsylvania 15213, United States

[b]Chemistry and Nanoscience Center, National Renewable Energy Laboratory, 15013 Denver West Parkway, Golden, Colorado, 80401, United States

[c]School of Applied and Engineering Physics, Cornell University, Ithaca, New York 14853, United States

[d]Department of Chemistry and Chemical Biology, Baker Laboratory, Cornell University, Ithaca, New York, 14853, United States

corresponding author email address: noonan@andrew.cmu.edu

Table of Contents

Materials and Methods S4-S8

Experimental Procedures S9-S13

Polymerization Kinetics

- **Table S1**. Mole fraction monomer in the feed for copolymerizations of NB-5-Hex and NB-5-BuBr S14
- **Table S2**. Mole fraction monomer converted to polymer after 5 minutes in copolymerizations of NB-5-Hex and NB-5-BuBr S14

Gas Chromatography Mass Spectrometry (GCMS)

- **Figure S1**. Stacked plot of gas chromatograms for conversion analysis of the 67:33 NB-5-Hex:NB-5-BuBr copolymerization S15
- **Figure S2**. Stacked plot of gas chromatograms for conversion analysis of the 50:50 NB-5-Hex:NB-5-BuBr copolymerization S16
- **Figure S3**. Stacked plot of gas chromatograms for conversion analysis of the 33:67 NB-5-Hex:NB-5-BuBr copolymerization S17

Percent Conversion

- **Figure S4**. Plot of percent conversion versus time for DP 500 copolymerizations of NB-5-Hex and NB-5-BuBr S18

Gel-Permeation Chromatography (GPC)

- **Figure S5**. GPC traces for PNB-Hex-Br copolymers after conversion analysis S18

Polymer Characterization

NMR Spectroscopy

- **Figure S6**. Stacked plot of ^{1}H NMR spectra for DP 500 PNB-Hex-Br copolymers S19
- **Figure S7**. Stacked plot of ^{1}H NMR spectra for DP 1000 and DP 2000 60:40 PNB-Hex-Br copolymers S20
- **Figure S8**. ^{1}H NMR spectra for DP 500 60:40 PNB-Hex-Br copolymer (top) and the corresponding PNB-Hex-NMe_3[Br] AEM after functionalization with trimethylamine (bottom) S21
- **Figure S9**. ^{1}H NMR spectra for DP 500 60:PNB-Hex-Br copolymer (top) and the corresponding PNB-Hex-iPrMe[PF_6] AEM after functionalization with $[N(iPr)Me]_3$P=N-Me (bottom) S22
- **Figure S10**. ^{31}P{^{1}H} NMR spectrum for 60:40 PNB-Hex-iPrMe[PF_6] copolymer S23

GPC

- **Figure S11**. GPC traces for PNB-Hex-Br copolymers used to make anion exchange membranes…………………………………………………..S24

Small Angle X-ray Scattering (SAXS)

- **Figure S12**. SAXS scattering patterns of statistical (left) and pentablock (right) 67:33 PNB-Hex-iPrMe[Cl] S24

Thermogravimetric Analysis (TGA)

- **Figure S13**. TGA traces for DP 500 ionic copolymers S25

Electrochemical Impedance Spectroscopy (EIS)

- **Figure S14**. Sample Nyquist Plots for 60:40 PNB-Hex-NMe_3[OH] S26

FUEL CELL DURABILITY AND HYDROGEN CROSSOVER

- Figure S15. Durability of the fuel cells and hydrogen crossover measurements for 60:40 PNB-Hex-NMe_3[OH] and 60:40 PNB-Hex-iPrMe S27

REFERENCES S28-S29

Materials and Methods

All commercially available chemicals were purchased and were used as received. Tri-*tert*-butylphosphine palladium (II) methyl chloride was synthesized according to a modified literature procedure.[1] 5-hexylbicyclo[2.2.1]hept-2-ene or 5-*n*-hexyl-2-norbornene (NB-5-Hex) and 5-(4-bromobutyl)bicyclo[2.2.1]hept-2-ene or 5-(4-bromobutyl)-2-norbornene (NB-5-BuBr) were prepared according to prior reports and both compounds were isolated as ~4:1 *endo*:*exo* mixtures.[2, 3] Tris(isopropyl-(methyl)amino)(methylamino)phosphonium hexafluorophosphate(V) was prepared according to literature procedures.[4, 5] All polymerizations were performed in dry, degassed CH_2Cl_2 (dried using a JC Meyer solvent system).

NMR Analysis. All NMR spectra were recorded on a 500 MHz Bruker Avance 3 Spectrometer or a 500 MHz Bruker Avance Neo with a Prodigy Cryoprobe. The ^{1}H NMR spectra of compounds and polymers were collected in either deuterated chloroform ($CDCl_3$) or deuterated tetrachloroethane (TCE-d_2) and referenced to residual protio solvents (7.26 ppm for $CHCl_3$ and 6.00 ppm for TCE-*d*). The ^{31}P NMR spectra were referenced to the lock signal.

Gas chromatography-Mass Spectrometry (GC-MS) Analysis. GC-MS analysis was performed on a Hewlett-Packard Agilent 6890-5973 GC-MS workstation. The GC column was a Restek fused silica capillary column (RTX-5). Helium was used as the carrier gas. The following conditions were used for all GC-MS analyses: injector temperature, 250 °C; initial temperature, 50 °C; temperature ramp, 10 °C/min; final temperature, 170 °C. For estimation of conversion, 0.05 mL aliquots were removed from polymerization reactions and quenched with 2 drops of acetonitrile in a scintillation vial. This was then diluted with 5 mL of ethyl acetate, which resulted in precipitation of the polymer from the solution. Once the polymer settled to the bottom of the

vial, the solution was then pipetted into a 2 mL vial for analysis. Conversion was calculated by comparison of the monomer integrations with the internal standard (1,3,5-trimethoxybenzene).

Gel-Permeation Chromatography (GPC). GPC measurements were performed on a Waters Instrument equipped with a 2690 autosampler, a Waters 2414 refractive index (RI) detector, and two SDV columns (Porosity 1000 and 100000 Å; Polymer Standard Services). The eluent tetrahydrofuran (THF) was doped with 10 mM lithium bis(trifluoromethanesulfonyl)imide (flow rate of 1 mL/min, 40 °C). A 9-point calibration based on polystyrene standards (Polystyrene, ReadyCal Kit, Polymer Standard Services) was applied for determination of molecular weights.

Thermogravimetric Analysis (TGA). TGA was carried out using a TA Instruments TGA Q50. Samples were heated from 50 – 800 °C at a rate of 10 °C/min under a N_2 atmosphere.

Conductivity, Ion Exchange Capacity (IEC), and Water Uptake (WU). All measurements were carried out similarly to prior reports.[4-6] Conductivity was measured by four probe electrochemical impedance spectroscopy (EIS) using a Scribner Membrane Conductivity Clamp and a Bio-Logic SP-150 Potentiostat.[4-6] IEC was measured using standard back titration methods.[4-6] Water uptake was determined gravimetrically using the following equation:

$$WU\ (\%) = \frac{Wet\ Membrane\ (\text{–}OH\ form) - Dry\ Membrane(\text{–}Cl\ form)}{Dry\ Membrane\ (\text{–}Cl\ form)} \times 100$$

Small Angle X-Ray Scattering (SAXS). Dried thin film membranes in the bromide or chloride form were mounted on a membrane sample holder. SAXS was performed using an Anton Paar SAXSess mc^2 with narrow-slit beam collimation.[5, 6]

Cryogenic Transmission Electron Microscopy (cryo-TEM). Sectioning of the PNB-Hex-NMe_3[Cl] and PNB-Hex-iPrMe[Cl] copolymers for cryo-TEM was performed using cryo-

microtomy on a Leica EM UC7/FC7 Cryo-Ultra-microtome. The samples were cooled to temperatures between −60 to −95°C and cut into ~30 nm thick sections using a diamond knife. These sections were then collected using copper TEM grids with lacey carbon support and stored at room temperature. Cryo-TEM images of the grids were taken on a Thermo Fisher Talos Arctica microscope operated at an accelerating voltage of 200keV and using a Gatan K3 direct electron detector. The microscope was operated at a temperature of −190 °C and in energy-filtered mode using a 20eV energy slit. Images for the PNB-Hex-NMe_3[Cl] and PNB-Hex-iPrMe[Cl] were recorded at defocus values between -1 and -5 µm.

Membrane Electrode Assembly (MEA) preparation. MEA fabrication was carried out similarly to prior reports with some minor modifications.[7-9] Two commercially available carbon-supported catalysts were selected as the electrocatalysts: platinum (Pt) supported on Vulcan carbon catalyst (40 wt.% Pt on carbon black, Alfa Aesar; denoted as Pt/C) was used at the cathode and platinum ruthenium (PtRu) supported on Vulcan carbon catalyst (40 wt.% Pt, 20 wt.% Ru on Vulcan XC72 carbon, Alfa Aesar; denoted as PtRu/C) was used at the anode. Catalyst ink dispersions were prepared by suspending Pt/C or PtRu/C into water and 2-propanol, where the water/2-propanol volume ratio is 1:10. A perfluorinated anion exchange material supplied by the National Renewable Energy Laboratory[10] was used as the ionomer at a target loading of 3 wt% in the catalyst ink dispersion. To fabricate the gas diffusion electrodes (GDEs), the catalyst ink was sprayed onto the gas diffusion layer with a SonoTek ultrasonic spray coating station. The gas diffusion layers (GDLs) were Toray paper (Toray Carbon paper 060 wet proofed) for the anode and Freudenberg H23C8 for the cathode. The target loading of anode and cathode electrodes were 0.8 mg_{PtRu}/cm^2 and 0.4 mg_{Pt}/cm^2, respectively. Previous fuel cell testing and optimization influenced the choice of gas diffusion layers. Before assembling the cell, GDEs and membranes

were soaked in 1 M KOH solution for 12 h at room temperature and then assembled while still slightly wet with KOH solution. The membrane was sandwiched between two GDEs with 5 cm^2 active area, PTFE gaskets were used and a GDL compression of 25% was targeted.

Fuel Cell Testing. The assembled MEA was attached to a modified Scribner Fuel Cell Test Station (890E). The cell temperature and the dew points of anode and cathode were set at 70 °C. H_2 and N_2 were supplied to the anode and cathode, respectively, at 0.5 standard liter per minute (slpm) until the desired temperature was achieved. Then, the N_2 was switched to O_2. The anode and cathode flow rates were increased to 1.0 slpm. Back pressure of 131 kPa was applied to the cell. After open circuit voltage (OCV) stabilized, a constant voltage of 0.5 V was applied until a plateau in the current density was observed. Polarization curves were measured by sweeping voltage from OCV to 0.1 V at a scan rate of 10 mVs^{-1}. High frequency resistance (HFR) was measured by single frequency (5.5 kHz) HFR measurement using the Scribner 880 loads. After collecting polarization data, the short-term durability was assessed for each MEA by holding current density at 600 mA/cm^2 and measuring the cell potential and HFR for a period (114 h for 60:40 PNB-Hex-NMe_3[OH], 72 h for 60:40 PNB-Hex-iPrMe[OH]). The Department of Energy has targeted alkaline membrane fuel cell performance of 0.6 V at 600 mA/cm^2 on H_2/air (CO_2 free, maximum pressure of 1.5 atm) at T > 60 °C with less than 10% voltage degradation.[11]

Hydrogen Crossover Measurements

Hydrogen crossover measurements were conducted by flowing H_2 at the anode and N_2 at the cathode with a flow rate of 0.2 slpm before and after the durability test with a cell operating temperature = 70 °C, back pressure = 131 kPa, and 100% relative humidity. The H_2 crossover current was measured by applying a voltage of 0.5 V across the cell to obtain H_2 crossover limiting

current density (iH_2). The Department of Energy has targeted that the maximum H_2 crossover current density for a novel electrolyte membrane to be less than 2 mA/cm^2 at the beginning of life.[11]

Water Limiting Current

Before measuring limiting current, 100% relative humidity H_2 and N_2 were supplied to anode and cathode, respectively at 500 ml/min until desired cell temperature (70 °C) was achieved. Then, the cathode flow was switched from N_2 to O_2. After OCV stabilized, a constant voltage of 0.5 V was applied until a plateau in the current density was observed. The limiting currents were measured with an applied anode flow rate of 500 mL/min and cathode flow rate of 200 ml/min. With the cathode relative humidity at 0% (dry), the cell potential was held at 0.4V for 5 min. Then the cell potential was scanned from 0.4 V to 0.15 V in 0.025 V steps and the current density was recorded after allowing the cell to stabilize for 100 s.

EXPERIMENTAL PROCEDURES

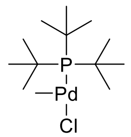

Tri-*tert*-butylphosphine palladium(II) methyl chloride (*t*Bu$_3$PPd(Me)Cl). The synthesis of this catalyst was carried out similarly to a literature report.[1] In a N_2 glovebox, tri-*tert*-butylphosphonium tetrafluoroborate (0.109 g, 0.376 mmol), 2 mL anhydrous CH_2Cl_2, and degassed triethylamine (0.05 mL, 0.36 mmol) were added to a 20 mL scintillation vial equipped with a stir bar and the reaction mixture was stirred for 30 min at room temperature to generate tri-*tert*-butylphosphine. Then, chloro(1,5-cyclooctadiene)methylpalladium(II) (0.100 g, 0.377 mmol) was added to the vial, and the mixture was stirred for 30 min at room temperature. The mixture was added to hexanes and the product precipitated as a yellow solid. The solid was isolated by vacuum filtration and recrystallized from 1:1 heptane:CH_2Cl_2 to afford bright yellow crystals. (0.092 g, 68% yield). NMR spectra matched with the previous report.[1]

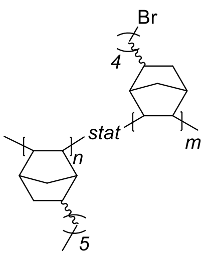

General Polymerization Procedure. All copolymers were synthesized in a similar manner with varied ratios of monomer and catalyst loading to achieve the targeted percent functionalization and molecular weight. Below is an example procedure for the PNB-Hex-Br (60:40) copolymer with a targeted degree of polymerization (DP) of 500 repeat units. In a N_2 filled glovebox, an oven-dried Schlenk flask equipped with a stir bar was charged with tetrakis(pentafluorophenyl)boron lithium ethyl etherate (0.023 g, 0.0264 mmol) and *t*Bu$_3$PPd(Me)Cl (0.0095 g, 0.0264 mmol) in dry CH_2Cl_2 (6.6 mL). The flask was sealed, removed from the glovebox, and stirred for 15 min to ensure catalyst activation. In a separate reaction flask, NB-5-Hex (1.41 g, 7.91 mmol) and NB-5-BuBr (1.21 g, 5.28 mmol) were combined in dry CH_2Cl_2 (59.1 mL). The monomer solution was then injected by syringe into the activated catalyst solution. The polymerization reaction was stirred, and 0.05 mL aliquots were removed periodically to ensure complete consumption of the two monomers using

^{1}H NMR spectroscopy (vinyl proton disappearance). Polymers were precipitated into a large excess of methanol, which yielded an off-white fibrous polymer that was filtered and dried for 17 h *in vacuo* (2.51 g, 96% yield). ^{1}H NMR (500 MHz, $CDCl_3$) δ ppm: 3.4 (br s, 2H), 2.6 – 0.3 (br, all aliphatic protons), 0.88 (br s, 3H). The ^{1}H NMR shifts for each statistical copolymer in the series were all nearly identical, but integrations vary according to target monomer ratios and are indicated below for diagnostic $-CH_2Br$ and $-CH_3$ signals. For the kinetic studies, copolymerizations were carried out with 1,3,5-trimethoxybenzene as an internal standard (10 mol % relative to the two starting monomers) and aliquots were removed periodically for analysis using GC-MS.

Yields for other polymer samples:

DP 500 PNB-Hex-Br (67:33) 92% yield. DP 500 PNB-Hex-Br (50:50) 94% yield. DP 1000 PNB-Hex-Br (60:40) 92% yield. DP 2000 PNB-Hex-Br (60:40) 93% yield.

Percent Incorporation of NB-5-BuBr in PNB-Hex-Br copolymers. Incorporation of NB-5-BuBr in the PNB-Hex-Br copolymers was determined as in our prior report.[6] The integrations of the $-CH_2Br$ signal from the bromobutyl chain and the $-CH_3$ signal from the hexyl chain were compared after careful baseline correction of the spectrum to determine the relative ratio of the two monomers. Typically, ~50 mg of the polymer sample was dissolved in ~1 mL $CDCl_3$ for this analysis (delay time = 2s). The integration for the methylene signal was set to 2 (corresponding to 1 repeat unit of NB-5-BuBr) and the integration value for the $-CH_3$ signal from the hexyl chain was then divided by 3 to determine the relative ratio of the two monomers. A sample calculation is shown in Figure S6. In the ^{1}H NMR spectrum for the 67:33, 60:40 and 50:50 copolymers (NB-5-Hex:NB-5BrBu), ratios of 6:2, 4.5:2 and 3:2 should be observed for the terminal $-CH_3$ as compared to $-CH_2Br$ signal.

Drop-casting of PNB-Hex-Br copolymers 150 mg of polymer was dissolved in 3 - 5 mL of $CHCl_3$. Upon complete dissolution, the solution was filtered through a 0.22 μm PTFE syringe filter onto a stainless-steel dish (diameter – 5 cm). The $CHCl_3$ evaporated over 1 h to afford a transparent freestanding film, which was removed from the dish by immersion in DI water. The polymer was then dried *in vacuo* to remove water and any other residual solvents.

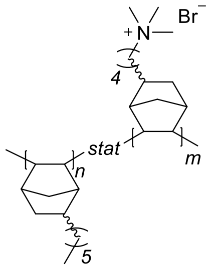

PNB-Hex-NMe3[Br] copolymers. The dried PNB-Hex-Br polymer films were immersed in an aqueous solution of 25% (w/v) trimethylamine for 48 h at room temperature. The solution was then replaced with fresh aqueous trimethylamine and the films were immersed for an additional 24 h. The films were removed and immersed in 3 × 100 mL portions of deionized water for 1 h each. The films were then dried *in vacuo* to afford the trimethylammonium-functionalized polymers.

^{1}H NMR (1:1 $CDCl_3$:CD_3OD) δ ppm: 3.2 - 2.95 (br, -CH_2N-), 2.85 (br s, -N(CH_3)$_3$), 2.4 – 0.2 (br, all other aliphatic *H*'s), 0.56 (br s, CH_3 of hexyl chain). The broad signal at 3.2 – 2.95 ppm is tentatively assigned to the methylene attached to nitrogen, but due to overlap with the solvent signal, it could not be integrated.

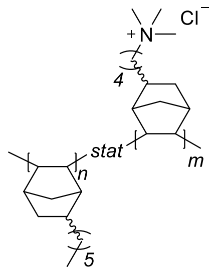

PNB-Hex-NMe3[Cl] copolymers. The PNB-Hex-NMe3[Br] copolymers were converted into the $^-$OH form by immersion of the thin films in 1 M KOH at 80 °C over a period of 48 h. The KOH solution was refreshed several times over the 48 h period, similar to prior reports.[6] The films were washed with deionized water 3 times for 20 min to remove residual KOH solution, and then the films were immersed in 0.1 M HCl solution to afford the Cl^- films.

PNB-Hex-iPrMe[PF_6] and [Cl] phosphonium copolymers.

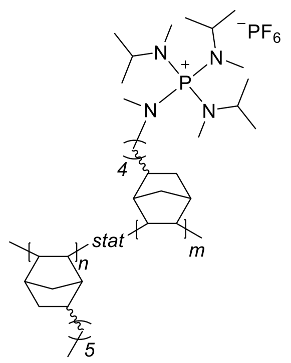

PNB-Hex-iPrMe[PF_6]. Synthesis was carried out similar to a prior report.[6] For the 60:40 PNB-Hex-Br, tris(isopropyl-(methyl)amino)(methylamino)phosphonium hexafluorophosphate(V) (1.19 g, 2.82 mmol) was dissolved in 1,2-dichlorobenzene (7.9 mL) and combined with 7.9 g of 50% (w/w) KOH_{aq} in a scintillation vial. The vial was heated to 60 °C for 30 min to deprotonate the N-H group and produce the neutral trisaminophosphazene ([N(*i*Pr)Me]$_3$P=N-Me). The water layer was removed, and then the organic phase was dried using Na_2SO_4 followed by filtration through celite. The resultant solution was combined with 350 mg of the PNB-Hex-Br copolymer (60:40) and placed in a heating block at 60 °C under an N_2 atmosphere in a glovebox for 48 h. The reaction mixture was then removed from the glovebox and precipitated from 100 mL KPF_6 saturated methanol. The precipitate was vacuum filtered and soaked in 20 mL KPF_6 saturated methanol at 22 °C (3 × 1 h). The precipitate was then vacuum filtered and dried under vacuum at 22 °C for 17 h to afford a white solid (0.420 g, 71% yield).

^{1}H NMR (500 MHz, TCE-d_2) δ ppm: 3.45 (br s, N-CH(CH_3)$_2$), 2.86 (br s, N-CH_2-), 2.70 (br s, N-CH_3), 2.58 (br s, N-CH_3), 2.35 – 0.4 (br, all other aliphatic *H*'s), 0.88 (br s, -CH_3). ^{31}P{^{1}H} NMR (202 MHz, TCE-d_2) δ 44.0 (s), –144.5 (septet, $^1J_{PF}$ = 714 Hz). Signal integrations will vary depending on relative ratio of the comonomers in the PNB-Hex-Br starting copolymer.

PNB-Hex-iPrMe[Cl]. PNB-Hex-iPrMe[PF_6] (0.420 g) was dissolved in a methanol:1,2-dichloroethane solution (50% v/v) and 5 g of a chloride ion exchange resin was added. The slurry was gently stirred for 17 h and, afterwards, the resin was removed using gravity filtration and the solution was concentrated using rotary evaporation. $^{31}P\{^{1}H\}$ NMR can be used to monitor the disappearance of the PF_6^- anion. If the exchange is incomplete, resin and solvent can be re-added to continue the exchange process. Yield of PNB-Hex-iPrMe[Cl] (60:40): 0.318 g, (87%). For the Cl^- form material, the ^{1}H spectrum is very similar to the PF_6^- form material and the $^{31}P\{^{1}H\}$ spectrum is also nearly identical except for the absence of the septet at −144.5 ($^{1}J_{PF}$ = 714 Hz) providing evidence of the displacement of the PF_6^- anion with Cl^-.

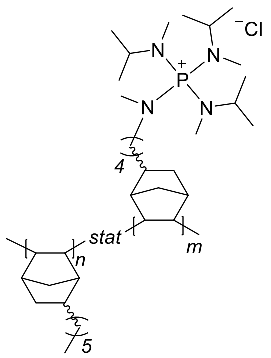

General procedure for drop-casting PNB-Hex-iPrMe[Cl]. 150 mg of polymer was dissolved in 3 mL of $C_2H_4Cl_2$. Upon complete dissolution, the solution was filtered onto a stainless-steel dish (diameter – 5 cm). The $C_2H_4Cl_2$ evaporated over ~8 h to afford a transparent freestanding film, which was removed from the dish by immersion in DI water. The polymer was then dried *in vacuo* to remove water and any other residual solvents.

Polymerization Kinetics

Table S1. Mole fraction of monomer in the feed prior to initiating copolymerization of NB-5-Hex and NB-5-BuBr as determined by GC-MS relative to the internal standard.

Target NB-5-Hex: NB-5-BuBr ratio	Target degree of polymerization	Mole fraction monomer in feed (t_0)			
		Endo NB-5-Hex	*Exo* NB-5-Hex	*Endo* NB-5-BuBr	*Exo* NB-5-BuBr
67:33	500	0.491	0.139	0.287	0.083
50:50	500	0.396	0.101	0.389	0.113
33:67	500	0.290	0.072	0.491	0.147

Table S2. Mole fraction monomer in polymer in copolymerizations NB-5-Hex and NB-5-BuBr after 5 minutes as determined by GC-MS (monomer consumption) relative to the internal standard.

Target NB-5-Hex: NB-5-BuBr ratio	Target degree of polymerization	Mole fraction monomer in polymer (t_{5min})			
		Endo NB-5-Hex	*Exo* NB-5-Hex	*Endo* NB-5-BuBr	*Exo* NB-5-BuBr
67:33	500	0.182	0.455	0.064	0.299
50:50	500	0.164	0.467	0.066	0.303
33:67	500	0.173	0.500	0.030	0.296

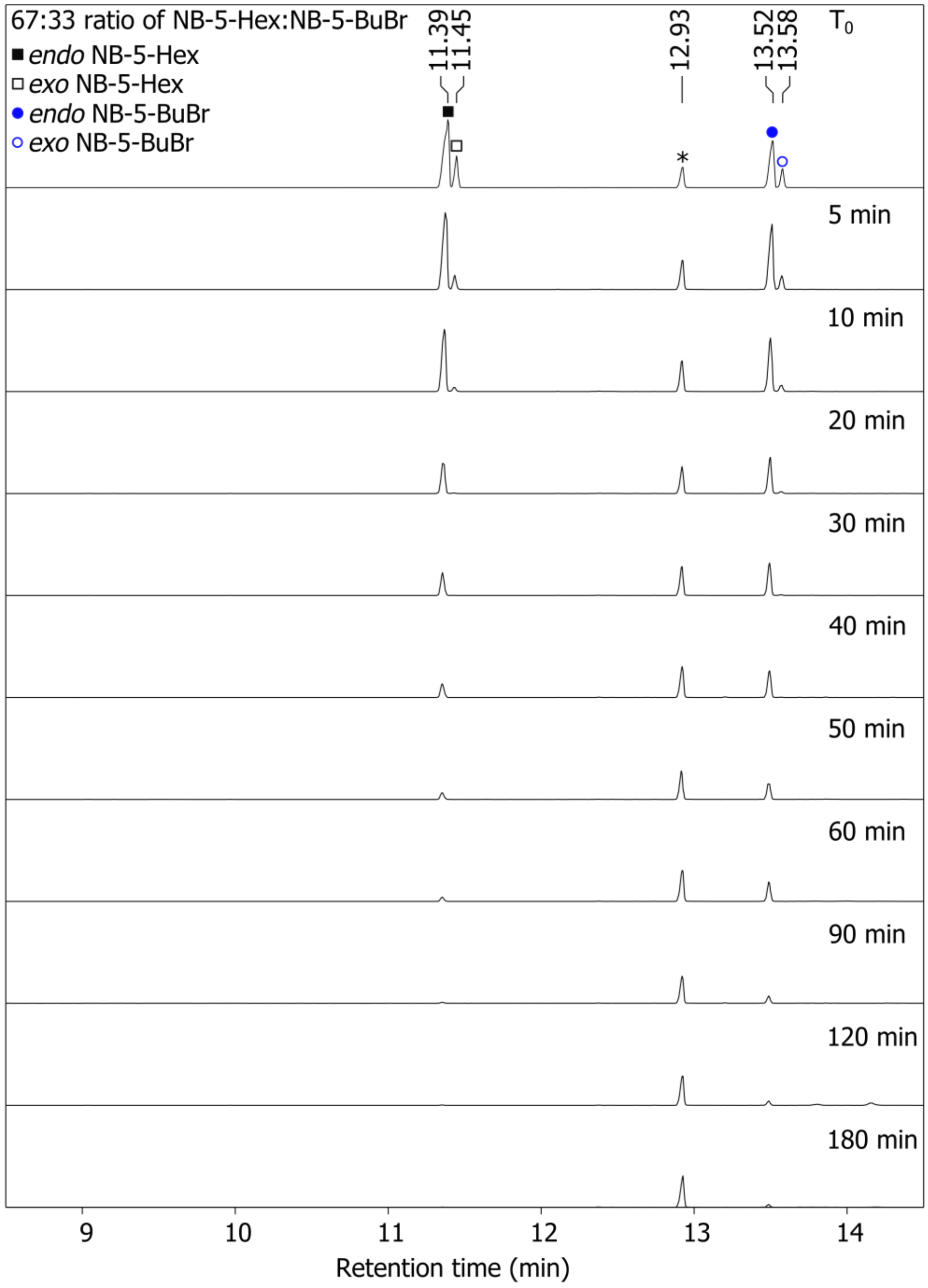

Figure S1. Stack plot of gas chromatograms for conversion analysis of the 67:33 PNB-Hex-Br copolymerization (* = 1,3,5-trimethoxybenzene). Semi-log plot of monomer consumption appears in Figure 1.

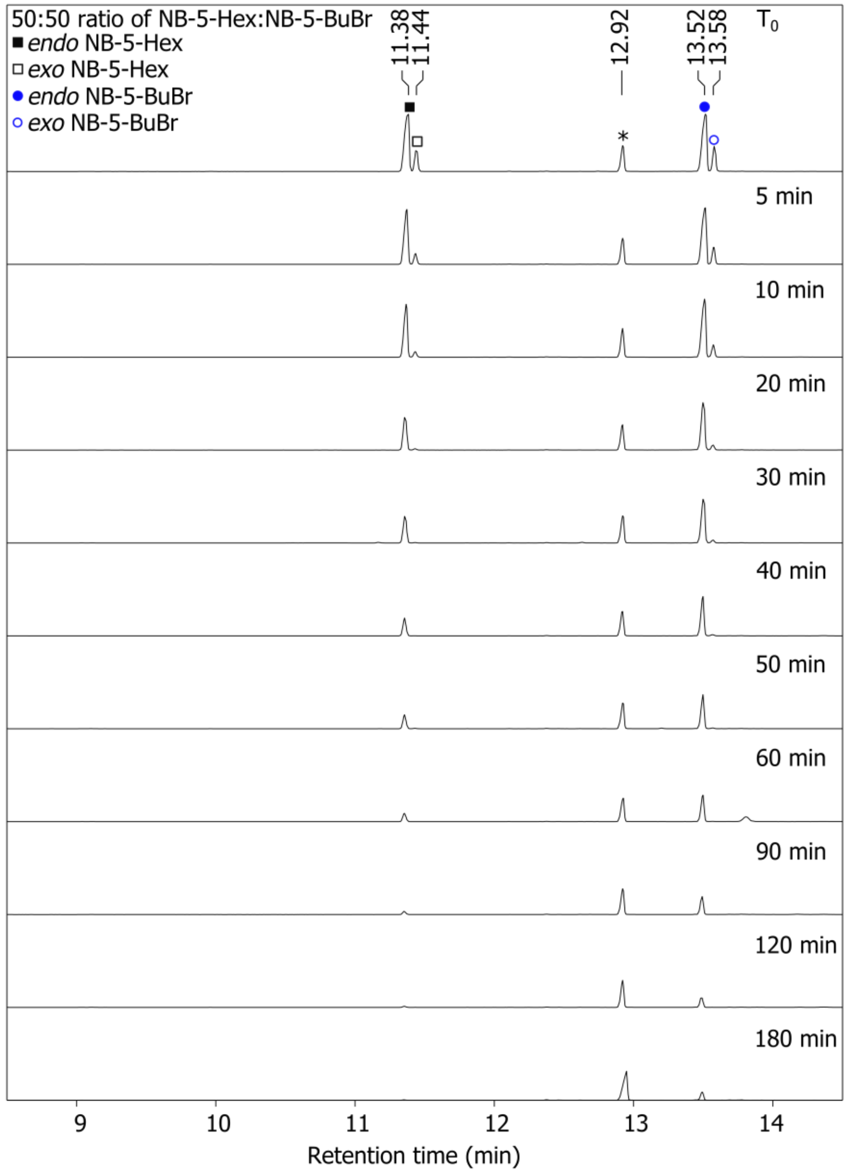

Figure S2. Stack plot of gas chromatograms for conversion analysis of the 50:50 PNB-Hex-Br copolymerization (* = 1,3,5-trimethoxybenzene). Semi-log plot of monomer consumption appears in Figure 1.

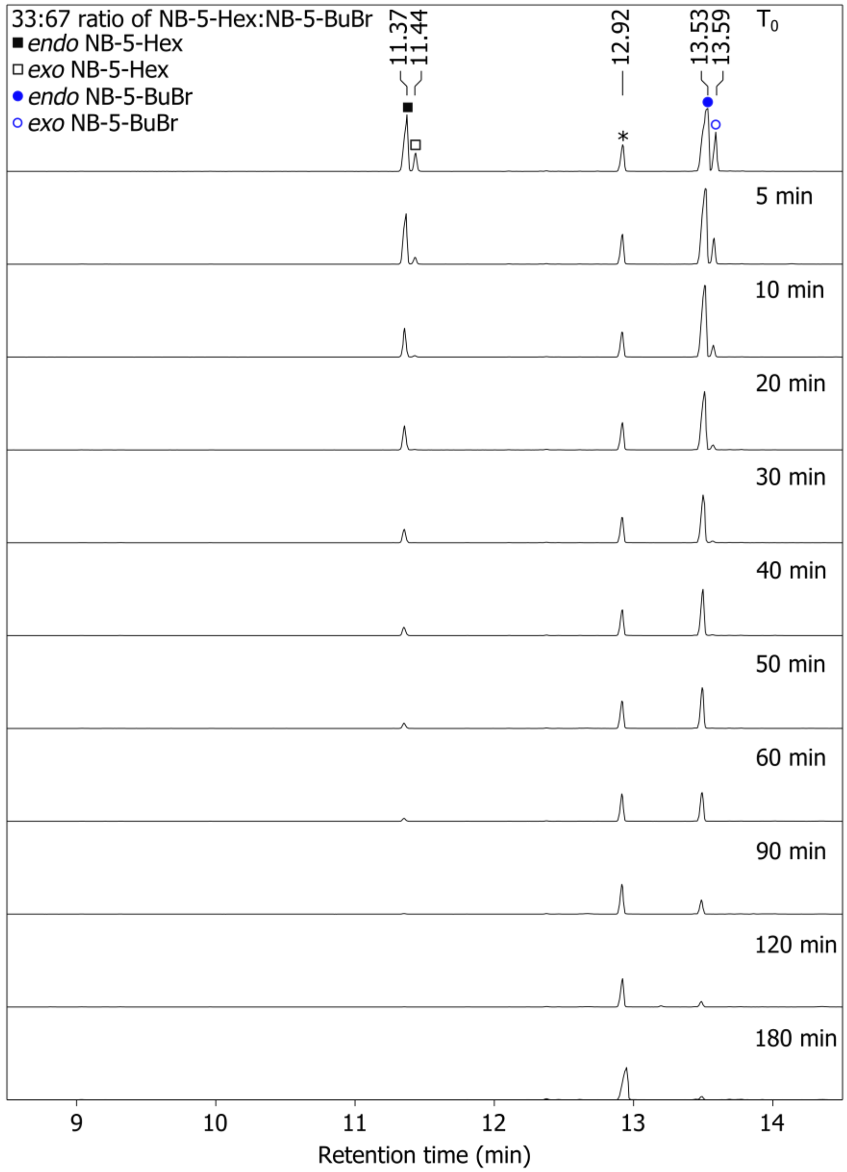

Figure S3. Stack plot of gas chromatograms for conversion analysis of the 33:67 PNB-Hex-Br copolymerization (* = 1,3,5-trimethoxybenzene). Semi-log plot of monomer consumption appears in Figure 1.

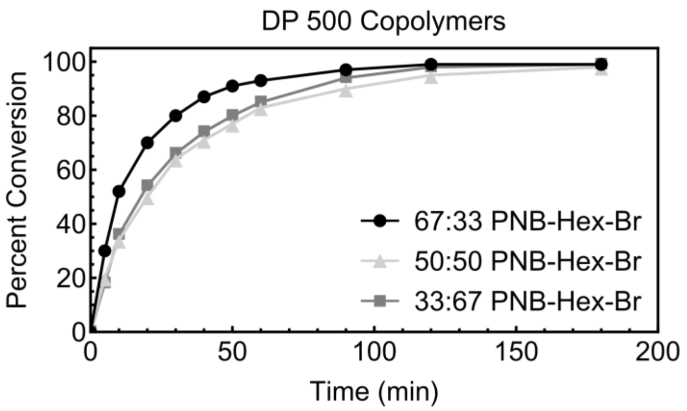

Figure S4. Percent monomer conversion vs. time plot for all DP 500 PNB-Hex-Br copolymers.

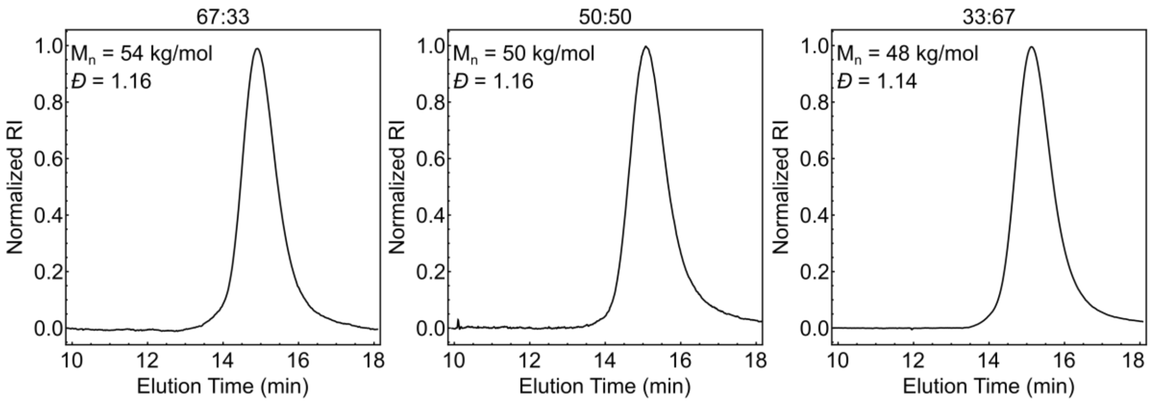

Figure S5. GPC traces for DP 500 PNB-Hex-Br copolymers after the final timepoint in conversion analysis.

POLYMER CHARACTERIZATION

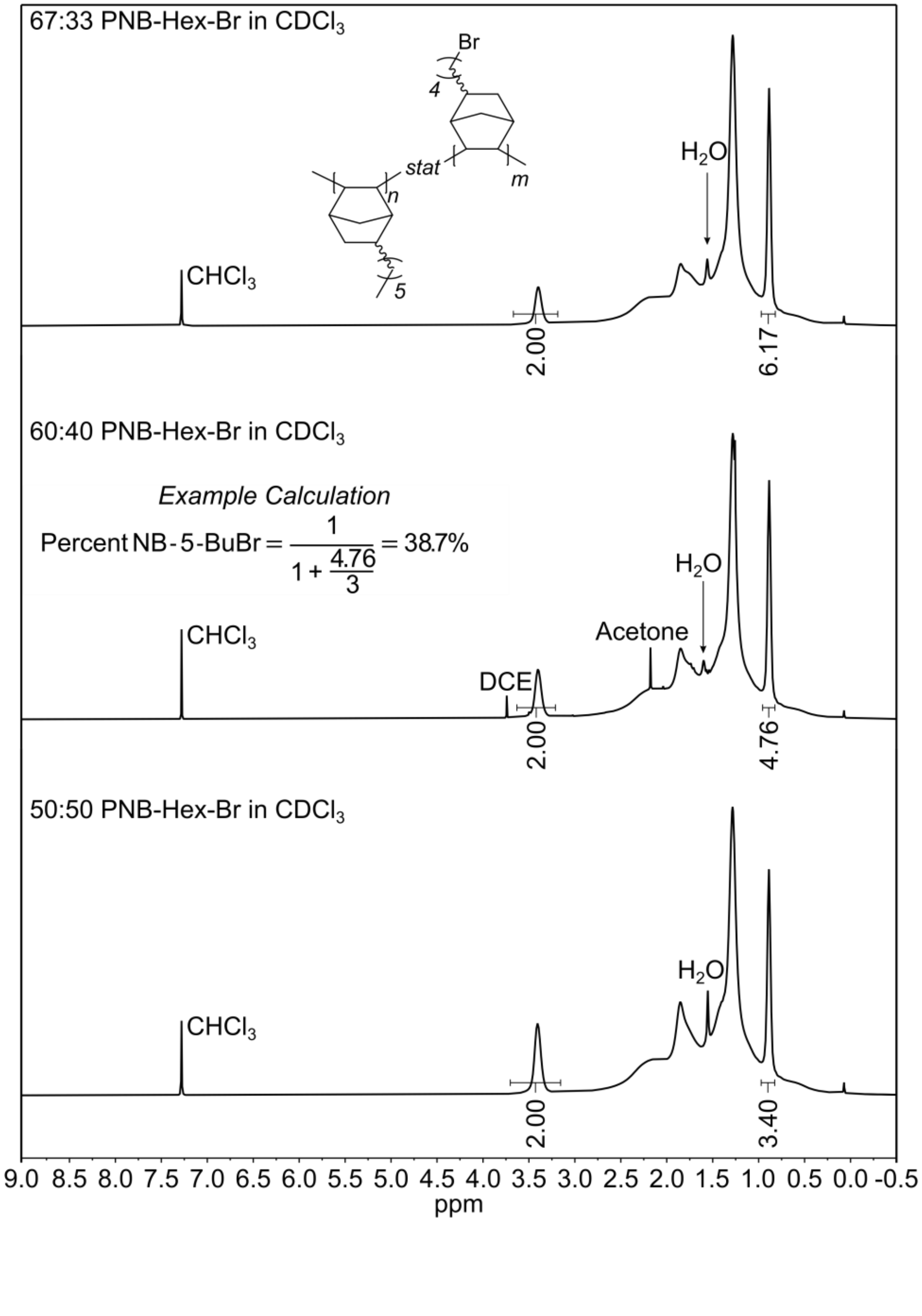

Figure S6. ^{1}H NMR spectra (500 MHz) of DP 500 PNB-Hex-Br copolymers (Top – 66:33, Middle – 60:40, Bottom – 50:50) collected in $CDCl_3$.

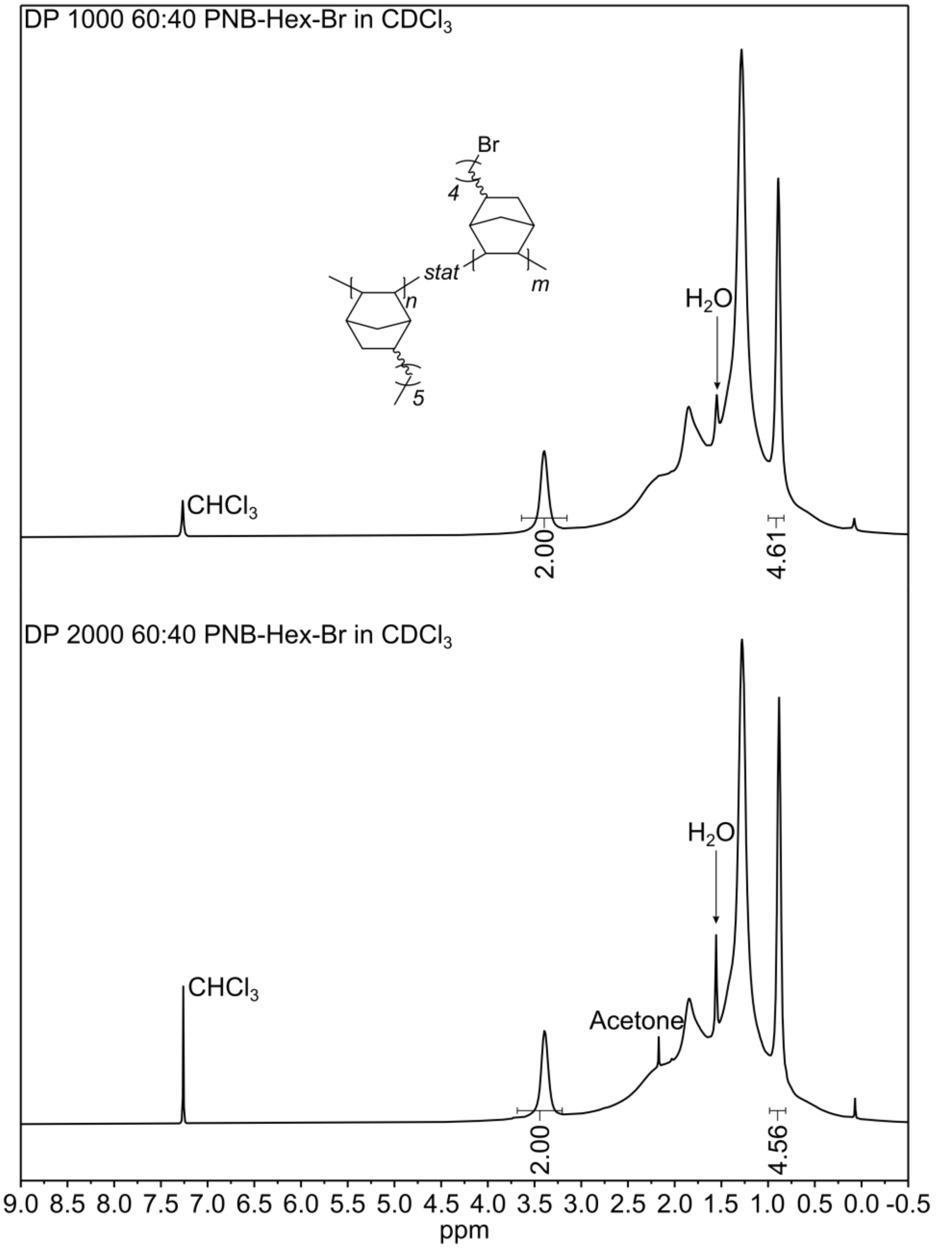

Figure S7. ^{1}H NMR spectra (500 MHz) of DP 1000 (Top) and DP 2000 (Bottom) PNB-Hex-Br copolymers collected in $CDCl_3$.

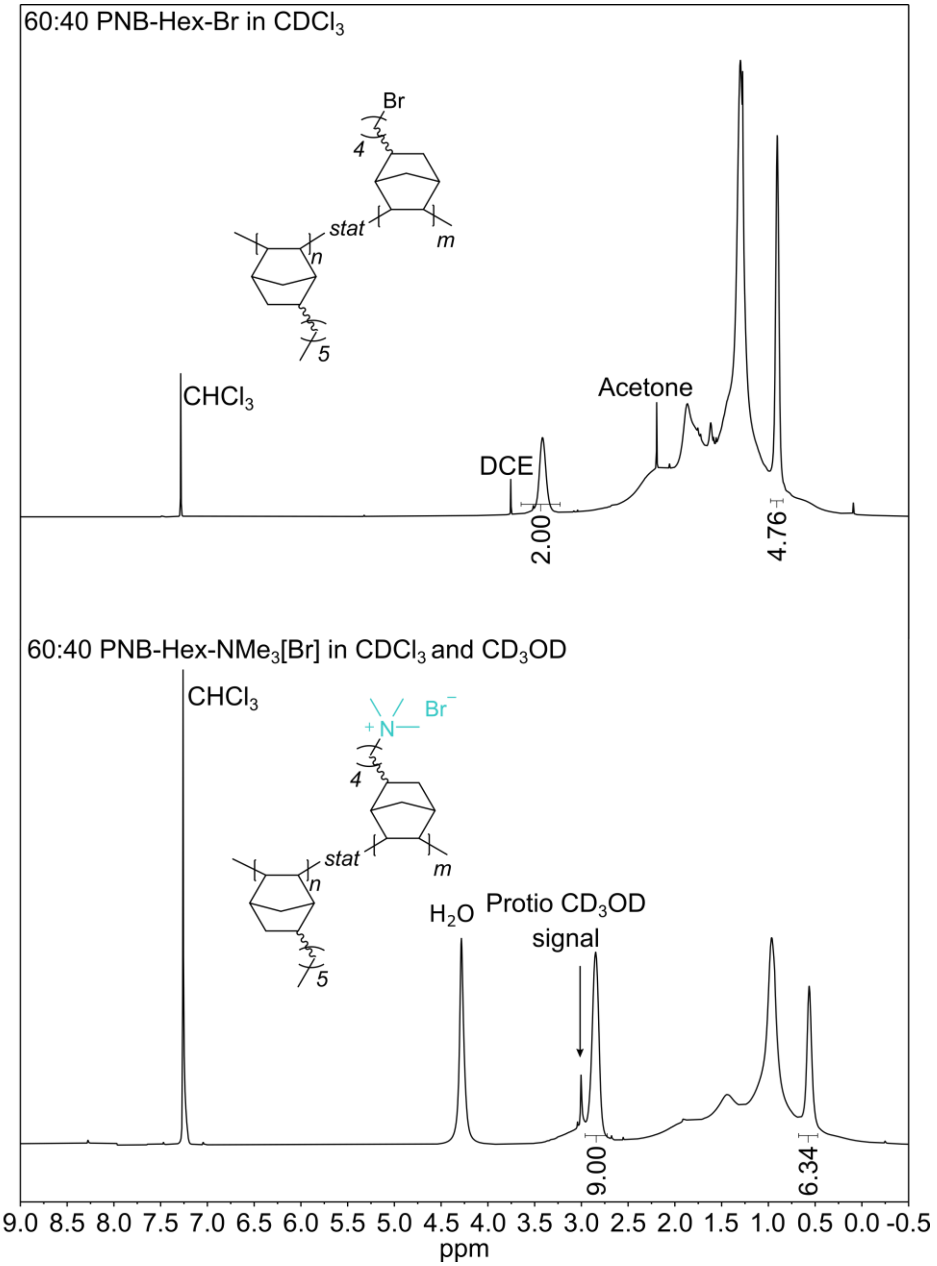

Figure S8. ^{1}H NMR spectra (500 MHz) of DP 500 60:40 PNB-Hex-Br copolymer collected in $CDCl_3$ (top) and the corresponding PNB-Hex-NMe_3[Br] polymer after functionalization with NMe_3 collected in 1:1 $CDCl_3$:CD_3OD (bottom). A 9:4.5 ratio should be observed for the N-methyl groups of the trimethylammonium to the terminal methyl group from NB-5-Hex for a 40 mol% ionic copolymer. Accurate integrals were difficult to obtain due to overlapping solvent and signal broadness.

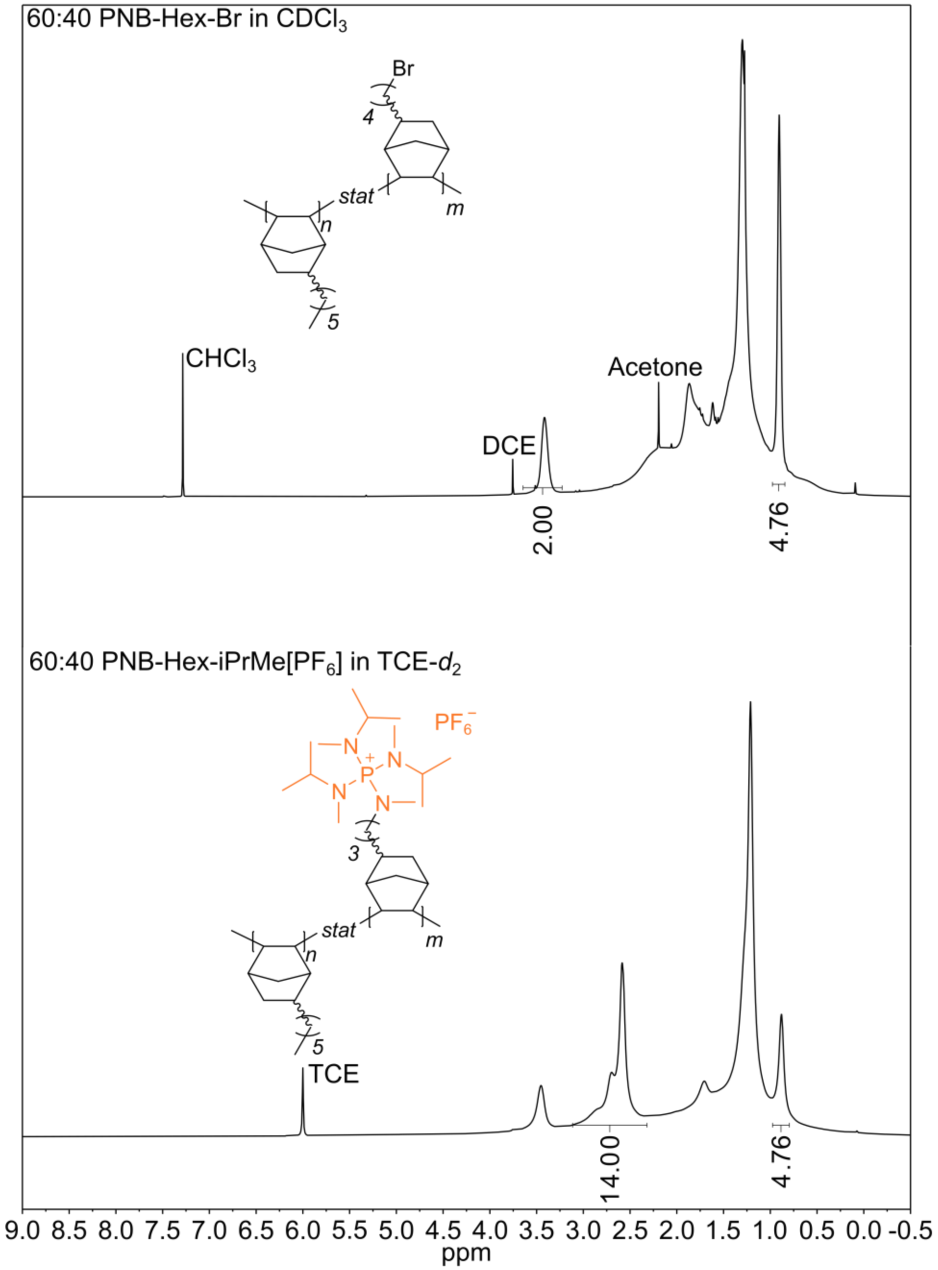

Figure S9. ^{1}H NMR spectra (500 MHz) of DP 500 60:40 PNB-Hex-Br copolymer collected in $CDCl_3$ (Top) and the corresponding PNB-Hex-iPrMe[PF_6] polymer after functionalization with $[N(iPr)Me]_3P$=N-Me collected in TCE-d_2 (Bottom). A 14:4.5 ratio should be observed for the N-methylene and N-methyl groups on the phosphonium relative to the terminal methyl group from NB-5-Hex for a 40 mol% ionic copolymer.

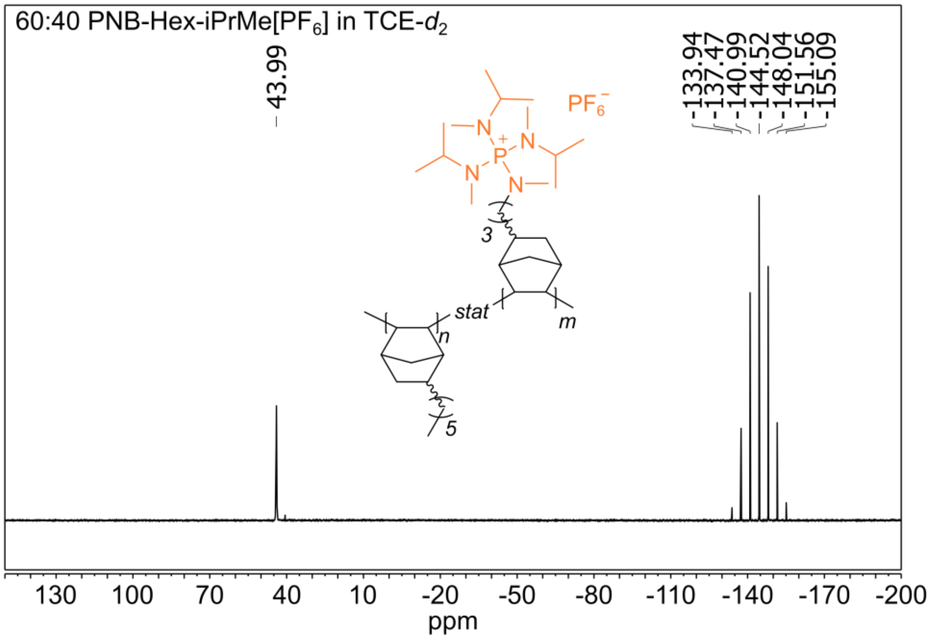

Figure S10. $^{31}P\{^{1}H\}$ NMR spectrum (202 MHz) of 60:40 PNB-Hex-iPrMe[PF_6] collected inTCE-d_2.

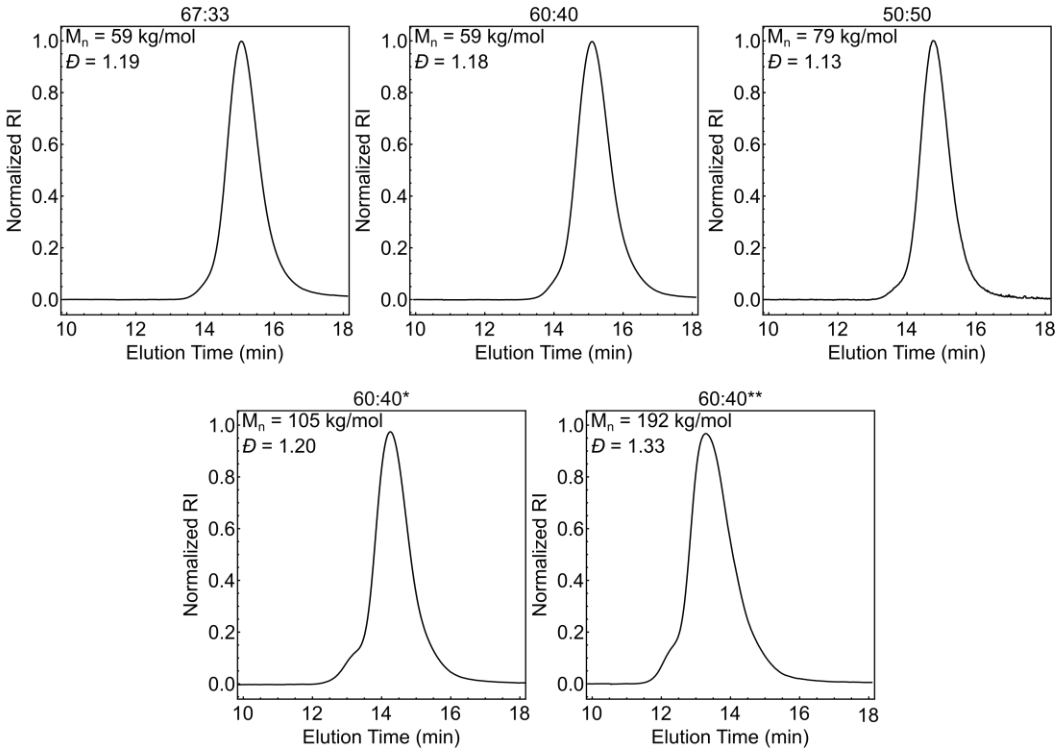

Figure S11. GPC traces for all PNB-Hex-Br copolymers. * Target degree of polymerization (DP) = 1000. ** Target DP = 2000. All other target DP's = 500.

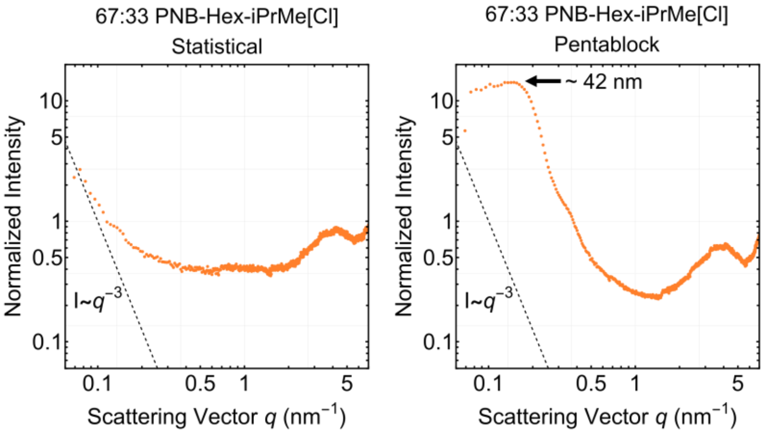

Figure S12. SAXS scattering patterns of statistical (left) and pentablock (right) 67:33 PNB-Hex-iPrMe[Cl] copolymers from our prior report.[6]

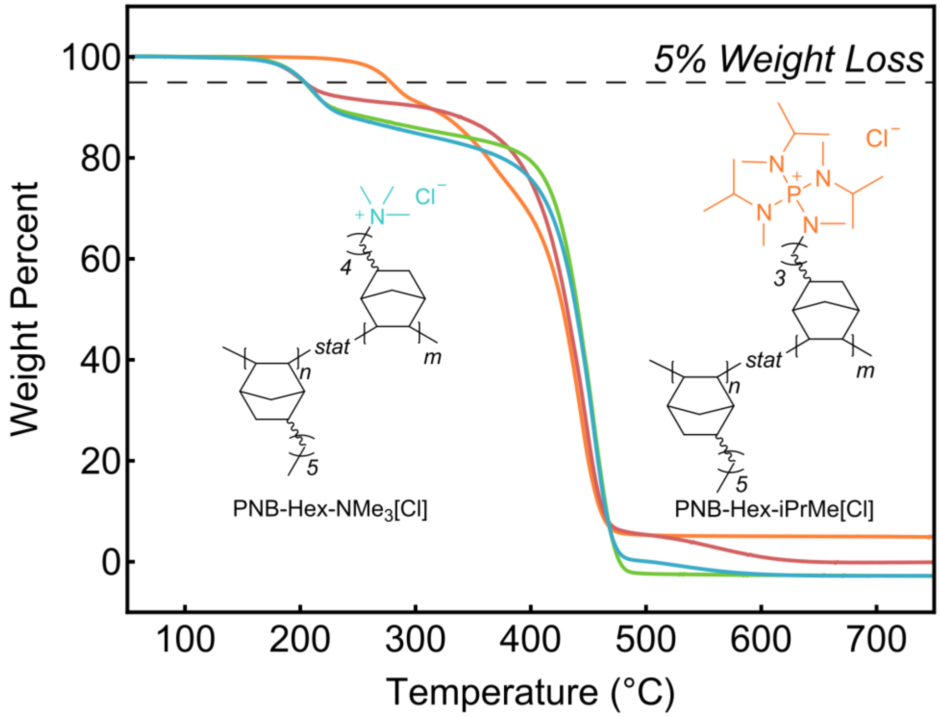

Entry	$T_{d5\%}$ (°C)
67:33 PNB-Hex-NMe_3[Cl]	204
60:40 PNB-Hex-NMe_3[Cl]	203
50:50 PNB-Hex-NMe_3[Cl]	203
60:40 PNB-Hex-iPrMe[Cl]	279

Figure S13. Thermogravimetric analysis traces for the PNB-Hex-NMe_3 and PNB-Hex-iPrMe copolymers in the Cl^- form (under N_2).

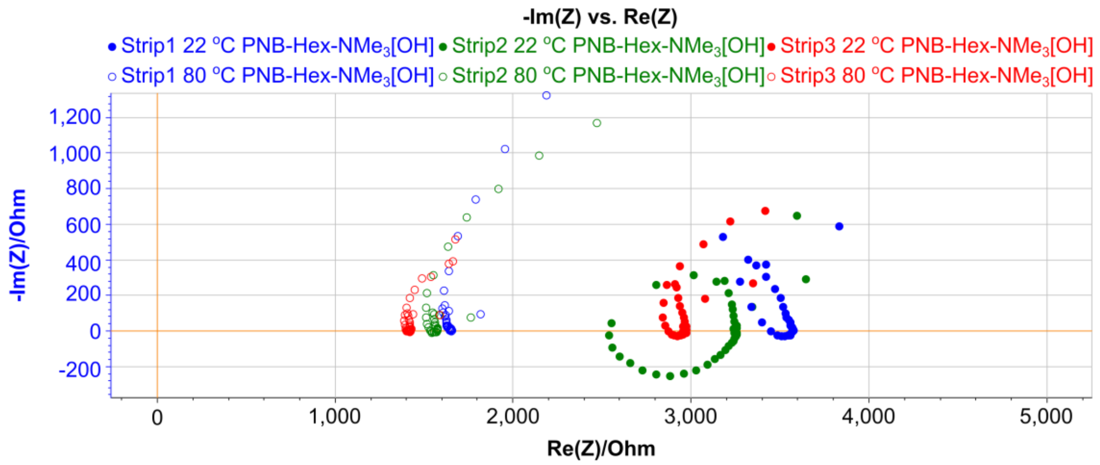

Entry	Width (cm)	Thickness (cm)	Ω 22 °C	Ω 80 °C	σ (⁻OH) 22 °C	σ (⁻OH) 80 °C
Strip1	0.55	0.0051	3569	1658	42.5	91.4
Strip2	0.60	0.0049	3262	1576	44.3	91.7
Strip3	0.60	0.0049	2959	1426	49.1	101.9

Figure S14. A set of sample impedance data for DP 500 60:40 PNB-Hex-NMe_3[OH] collected at 22 °C and 80 °C.

Sample calculation for Strip1:

$$\sigma\left(\frac{mS}{cm}\right) = \frac{L}{Z'WT} \times 1000$$

L is the length between electrodes = 0.425 cm, Z' is the real value of impedance = 3569 Ohms, W is the average width of the film = 0.55 cm, and T is the average thickness of the film = 0.0051 cm.

$$\sigma\left(\frac{mS}{cm}\right) = \frac{0.425\ cm}{3569\ Ohms\ \times\ 0.55\ cm \times 0.0051\ cm} \times 1000\ \frac{mS}{S} = 42.5\frac{mS}{cm}$$

FUEL CELL DURABILITY AND HYDROGEN CROSSOVER

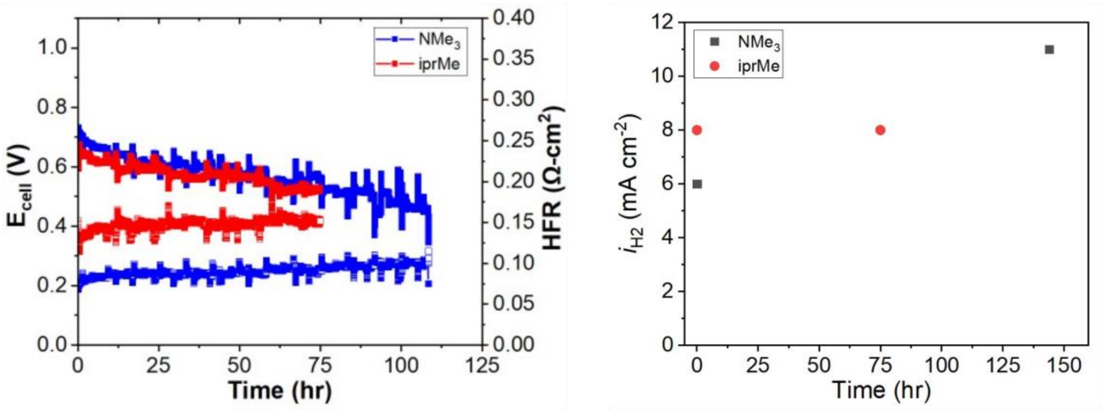

Figure S15. Left – Durability of the cells with 60:40 PNB-Hex-NMe_3[OH] (blue) and 60:40 PNB-Hex-iPrMe[OH] (red) Right – Hydrogen crossover measurements for 60:40 PNB-Hex-NMe_3[OH] (black square) and 60:40 PNB-Hex-iPrMe[OH] (red circle).

REFERENCES

(1) Yamashita, M.; Takamiya, I.; Jin, K.; Nozaki, K. Syntheses and Structures of Bulky Monophosphine-Ligated Methylpalladium Complexes: Application to Homo- and Copolymerization of Norbornene and/or Methoxycarbonylnorbornene. *Organometallics* **2006**, *25*, 4588-4595. DOI: 10.1021/om060347w.

(2) Pierre, F.; Commarieu, B.; Tavares, A. C.; Claverie, J. High T_g Sulfonated Insertion Polynorbornene Ionomers Prepared by Catalytic Insertion Polymerization. *Polymer* **2016**, *86*, 91-97. DOI: 10.1016/j.polymer.2016.01.047.

(3) Martínez-Arranz, S.; Albéniz, A. C.; Espinet, P. Versatile Route to Functionalized Vinylic Addition Polynorbornenes. *Macromolecules* **2010**, *43*, 7482-7487. DOI: 10.1021/ma101137z.

(4) Treichel, M.; Womble, C. T.; Selhorst, R.; Gaitor, J.; Pathiranage, T. M. S. K.; Kowalewski, T.; Noonan, K. J. T. Exploring the Effects of Bulky Cations Tethered to Semicrystalline Polymers: The Case of Tetraaminophosphoniums with Ring-Opened Polynorbornenes. *Macromolecules* **2020**, *53*, 8509-8518. DOI: 10.1021/acs.macromol.0c00422.

(5) Gaitor, J. C.; Treichel, M.; Kowalewski, T.; Noonan, K. J. T. Suppressing Water Uptake and Increasing Hydroxide Conductivity in Ring-Opened Polynorbornene Ion-Exchange Materials via Backbone Design. *ACS Appl. Polym. Mater.* **2022**, *4*, 8032-8042. DOI: 10.1021/acsapm.2c00297.

(6) Selhorst, R.; Gaitor, J.; Lee, M.; Markovich, D.; Yu, Y.; Treichel, M.; Gallegos, C. O.; Kowalewski, T.; Kourkoutis, L. F.; Hayward, R. C.; Noonan, K. J. T. Multiblock Copolymer Anion-Exchange Membranes Derived from Vinyl Addition Polynorbornenes. *ACS Appl. Energy Mater.* **2021**, *4*, 10273-10279. DOI: 10.1021/acsaem.1c02094.

(7) He, C.; Yang-Neyerlin, A. C.; Pivovar, B. Water Limiting Current Measurements in Anion Exchange Membrane Fuel Cells (AEMFCs); Part 1: Water Limiting Current Method Development. *J. Power Sources* **2022**, *539*, 231534. DOI: 10.1016/j.jpowsour.2022.231534.

(8) Yang-Neyerlin, A. C.; Medina, S.; Meek, K. M.; Strasser, D. J.; He, C.; Knauss, D. M.; Mustain, W. E.; Pylypenko, S.; Pivovar, B. S. Editors' Choice—Examining Performance and Durability of Anion Exchange Membrane Fuel Cells with Novel Spirocyclic Anion Exchange Membranes. *J. Electrochem. Soc.* **2021**, *168*, 044525. DOI: 10.1149/1945-7111/abf77f.

(9) He, C.; Yang-Neyerlin, A. C.; Pivovar, B. S. Investigating the Impact of the Ionomer on Alkaline Membrane Fuel Cell (AEMFC) Electrode Performance. *ECS Meet. Abstr.* **2021**, *MA2021-02*, 1055. DOI: 10.1149/MA2021-02361055mtgabs.

(10) Park, A. M.; Owczarczyk, Z. R.; Garner, L. E.; Yang-Neyerlin, A. C.; Long, H.; Antunes, C. M.; Sturgeon, M. R.; Lindell, M. J.; Hamrock, S. J.; Yandrastis, M. A.; Pivovar, B. S. Synthesis and Characterization of Perfluorinated Anion Exchange Membranes. *ECS Trans.* **2017**, *80*, 957-966. DOI: 10.1149/08008.0957ecst.

(11) *US Department of Energy, Fuel Cell Technical Team Roadmap*. 2017. https://www.energy.gov/eere/vehicles/downloads/us-drive-fuel-cell-technical-team-roadmap (accessed.
